# Functionalized Organosolv Lignins Suitable for Modifications of Hard Surfaces

**DOI:** 10.1021/acssuschemeng.0c00886

**Published:** 2020-04-20

**Authors:** Paola Giannì, Heiko Lange, Claudia Crestini

**Affiliations:** † Department of Chemical Sciences and Technologies, University of Rome “Tor Vergata”, Via della Ricerca Scientifica, 00133 Rome, Italy; ‡ Department of Pharmacy, University of Naples “Federico II”, V ia Domenico Montesano 49, 80131 Naples, Italy; § CSGI—Center for Colloid and Surface Science, Via della Lastruccia 3, 50019 Sesto Fiorentino, Italy; ∥ Department of Molecular Science and Nanosystems, University of Venice Ca’ Foscari, Via Torino 155, 30170 Venice Mestre, Italy

**Keywords:** organosolv lignin, lignin valorization, functionalization, enzymes, block-copolymers, ^31^P NMR

## Abstract

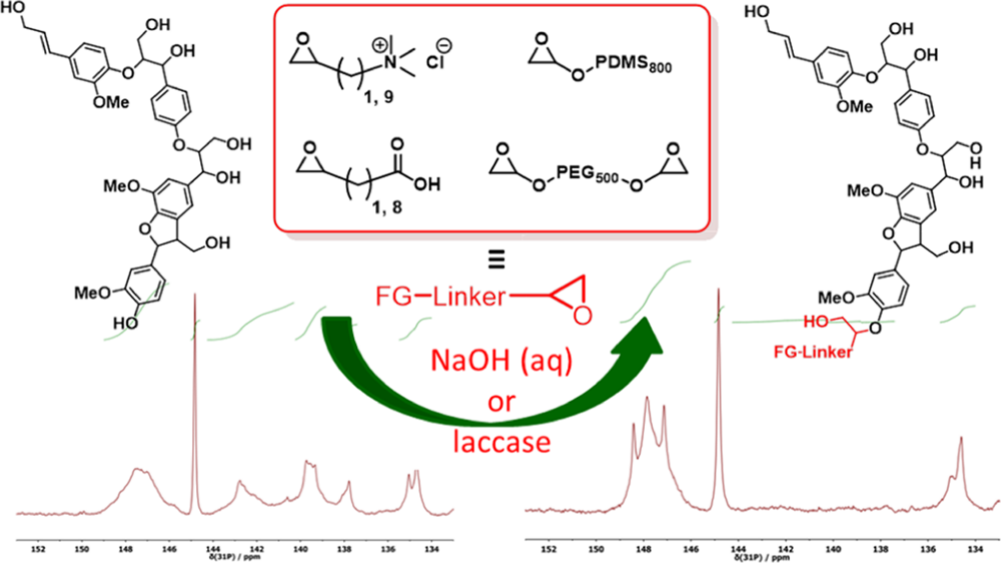

Two different organosolv lignins (OSLs), that is, wheat straw and corn stover OSLs, were chemically and enzymatically functionalized. Functional groups were attached via the formation of stable ether bonds exploiting the reactivity of free phenolic OH groups along the lignin backbone. The functional groups introduced a range from compact charged and chargeable building blocks for the generation of surface-active lignins to oligomeric and polymeric species used in lignin block-copolymer productions. Combination of selected functions led to novel charged or chargeable polymeric lignin-based materials. Products could be realized with different degrees of technical loadings in terms of introduced functional groups.

## Abstract 2

Industrially produced wheat straw and corn stover organosolv lignins were chemically and enzymatically functionalized via formation of ether bonds using various surface-active functionals.

## Introduction

Abundant polyphenolic nonfossil-based, but renewable resources continue to have problems in benefitting from the growing trends of sustainability in general and substitution of non-sustainable “traditional” active ingredients in everyday consumer products in particular.^1−3^ Especially lignin, despite enormous research efforts, still suffers from its intrinsic diversities and variabilities ranging from differences stemming from natural origins to issues emerging during industrially feasible isolations. Concerted efforts lead to ever improved processes allowing isolation of lignins with less purities^4−8^ or directly fractionating lignins into homogeneous components.^9−11^ As a direct consequence, now a detailed structural characterization of lignin is possible^12−18^ that can directly trigger the rational design of chemical functionalization/valorization strategies. Novel lignins and/or newly refined lignins thus need to be tested in eventually extended fields of application or retested within more focused fields of application.

Functionalization of novel industrially isolated lignins has the objective to change or improve their inherent characteristics and performances for making them suitable sustainable materials for specific downstream applications^1−3,19^ or for dedicated downstream processing. These modifications require control of lignin multifunctionality and are often run using simple and simplest chemistries^1,3,20−22^ or sustainable enzyme-based processes,^23−28^ within which de facto only the laccase-based ones have yet managed to bridge the gap between laboratory or pilot-scale applications and real-life usage.^29^

In this context, the present study screened several synthetic and biosynthetic approaches based on utilization and manipulation of phenolic groups in organosolv lignin (OSL) in order to arrive at a portfolio of fully functionalized and characterized lignins with specific tailored solubility and hydrophobicity characteristics. The development of a rigorous protocol for general chemical modification of lignin can only be prescient from a thorough structural characterization and deep knowledge of the specific lignin chemistry.

**Figure 1:**
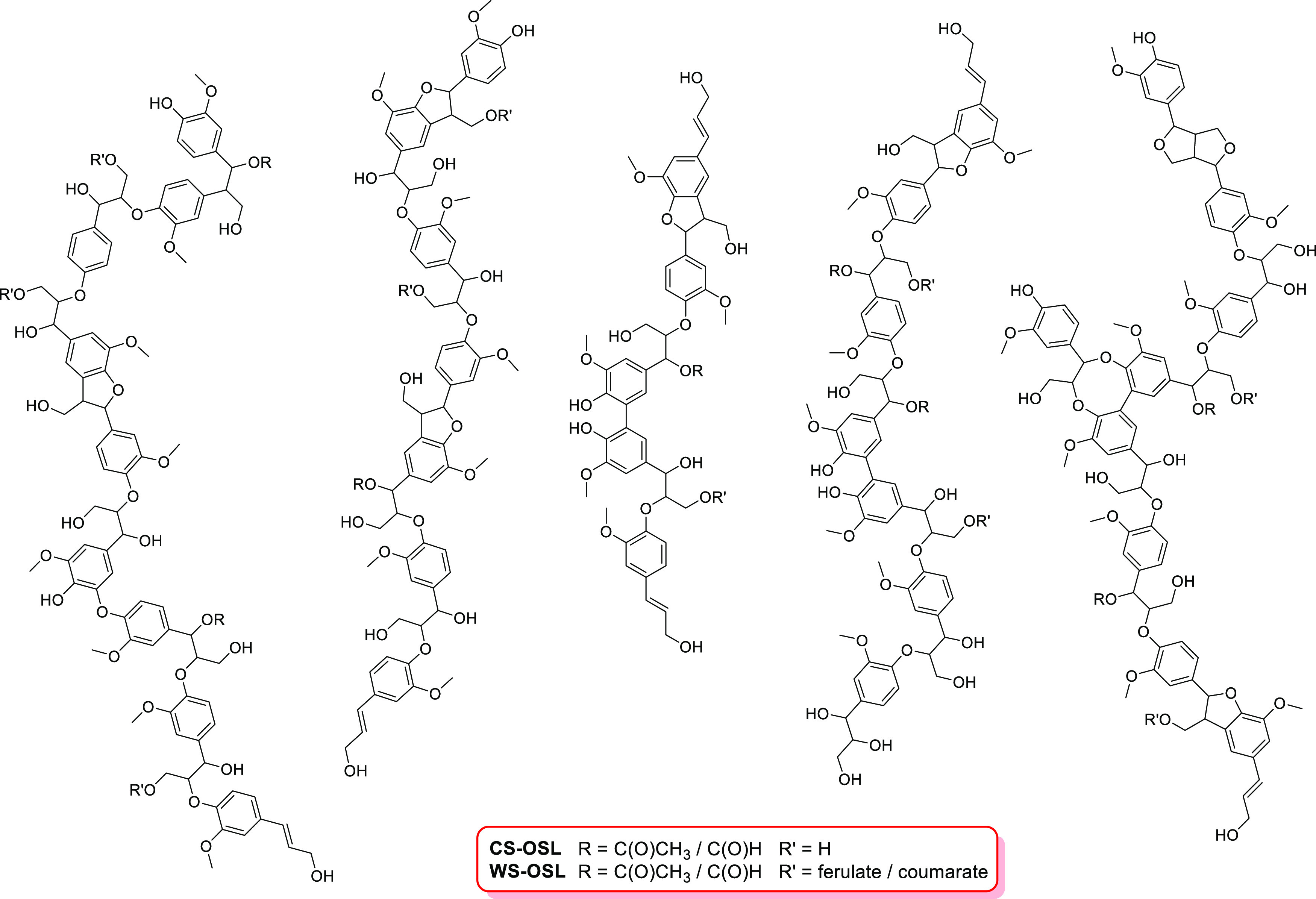
Structural features of OSLs, WS-OSL and CS-OSL. NB: the structures intend to give a conceptual overview of generally present groups; abundancies are not representative.

Starting from this prerequisite, two OSLs, namely wheat straw OSL, WS-OSL, and corn stover OSL, CS-OSL (Figure 1) produced via the CIMV organosolv biorefinery process^30^ were selected for this study. A detailed discussion regarding the fractionation of the very same material by fractional precipitation, including a detailed structural discussion of the fractions obtained, has been recently accomplished.^31^ This lignin was also used in an explorative study regarding the use of non-lignocellulolytic enzymes for lignin derivatisation.^32^ In the present effort, with the objective of maintaining the intrinsic polarity of the lignins used, strategies for functionalization were envisaged to achieve such functionalization without a net consumption of hydroxyl groups. Surface-active groups including permanently charged units, chargeable units, lipo- and hydrophilicity-enhancing motifs as well as combinations thereof were generally realized by attaching a functional motif to the lignin backbone via a linking unit by different sustainable chemical and biotechnological approaches.

## Materials and Methods

### General

WS-OSL and CS-OSL were produced via the Biolignin process by CIMV (Compagnie Industrielle de la Matière Végétale), Levallois Perret, France.^30^ Starting materials for the generation of functional groups and the generation of reactants, buffer salts, and solvents in appropriate grades were purchased from Sigma-Aldrich and used as received, if not stated otherwise. A 0.1 M aqueous acetate buffer solution at pH 5.0 was prepared freshly on a weekly basis. The non-commercially available reactants used for the functionalization of OSLs were synthesized following and/or adopting literature protocols: (i) *N*,*N*,*N*-trimethyl-9-(oxiran-2-yl)nonan-1-aminium chloride starting from 10-undecenyl chloride;^33,34^ (ii) 2-(oxiran-2-yl)acetic acid starting from 3-butenoic acid;^33^ and (iii) 9-(oxiran-2-yl)nonanoic acid starting from 10-undecanoic acid.^33^

Preparation of laccase (LAC) from *Trametes versicolor* in powder form was purchased from Sigma-Aldrich and used without further purification after determination of the actual enzymatic activity according to a literature procedure.^35^ In brief, LAC activity was determined spectrophotometrically using 2,2′-azino-bis-(3-ethylbenzthiazoline-6-sulphonate) (ABTS) as the substrate. The assay mixture contained 0.2 mM ABTS, 0.1 M sodium acetate at pH 5, and a suitable amount of enzyme, estimated on the basis of the activity given by the supplier; the substrate oxidation was followed by an absorbance increase at λ = 420 nm for 1 min (ε = 3.6 × 10^4^ m^–1^ cm^–1^).

### Chemical Functionalization of OSLs

Typically, OSL (500 mg) is dispersed in 10 mL of water and a volume of 1.0 M aqueous sodium hydroxide (NaOH) corresponding to 1 equiv of the total phenolic hydroxyl and carboxylic acid groups present in the OSL (as determined by quantitative ^31^P nuclear magnetic resonance (NMR) spectroscopy detailed below) is added. The overall reaction volume was subsequently adjusted to be 20 mL prior to addition of the functional. After 1 h of stirring at approx. 50 °C, the functional (see main text for actual examples), dissolved in 5 mL of distilled water, is added dropwise by means of a syringe pump over a time-span of 30 min in concentrations depending on the desired final technical loading. The reaction mixture is stirred at approx. 50 °C for additional 4–12 h. In order to assure appropriate mixing of lignin and functional in the reaction mixture in case of polydimethylsiloxane (PDMS)-based polymeric reactants, lauryl heptaethoxylate as a nonionic surfactant is used at a concentration of 5% (v/v).

### Enzyme-mediated Functionalization of OSLs

In a typical reaction, 500 mg of OSL is placed together with a determined amount of enzyme, typically 100 U, in 100 mL of 0.1 M aqueous acetate buffer in an Erlenmeyer flask and stirred for 15–24 h at approx. 50 °C after addition of the functional (see main text for actual examples) for derivatization. In order to assure appropriate mixing of lignin and functional in the reaction mixture in case of PDMS-based polymeric reactants, lauryl heptaethoxylate as the nonionic surfactant is used at a concentration of 5% (v/v).

### Isolation of Derivatized OSLs

After cooling to room temperature and acidifying to pH 2 using 10% (v/v) aqueous hydrogen chloride (HCl) solution, the resulting suspension is centrifuged (15 min at 5000 rpm) to recover the precipitated functionalized lignin; when needed, precipitation was forced by addition of concentrated sodium chloride solution. The precipitated functionalized lignin was then washed three to five times with 50 mL of acidified water (pH 2) followed by renewed isolation via centrifugation (15 min at 5000 rpm) each time. The final pellet was subsequently freeze-dried. The freeze-dried material is used for analysis and application without any additional manipulation if not stated otherwise.

### ^31^P NMR Analysis

In general, a procedure similar to the one originally published and previously applied was used:^36−38^ Approx. 30 mg of lignin were accurately weighed for analysis after phosphitylation using an excess of 2-chloro-4,4,5,5-tetramethyl-1,3,2-dioxa-phospholane (Cl-TMDP). ^31^P NMR spectra were recorded on a Bruker 300 MHz or Bruker 700 MHz NMR spectrometer controlled by TopSpin software, using an inverse gated decoupling technique with the probe temperature set to 20 °C. The maximum standard deviation of the reported data is 0.02 mmol g^–1^, whereas the maximum standard error is 0.01 mmol g^–1^.^36,39^ NMR data were processed with MestreNova (Version 8.1.1, Mestrelab Research). Technical loadings are determined by comparing the abundancies of total aromatic hydroxyl groups of the product lignin with the starting lignin.

### ^1^H NMR Analysis: Small Molecule Analysis

**Scheme 1:**
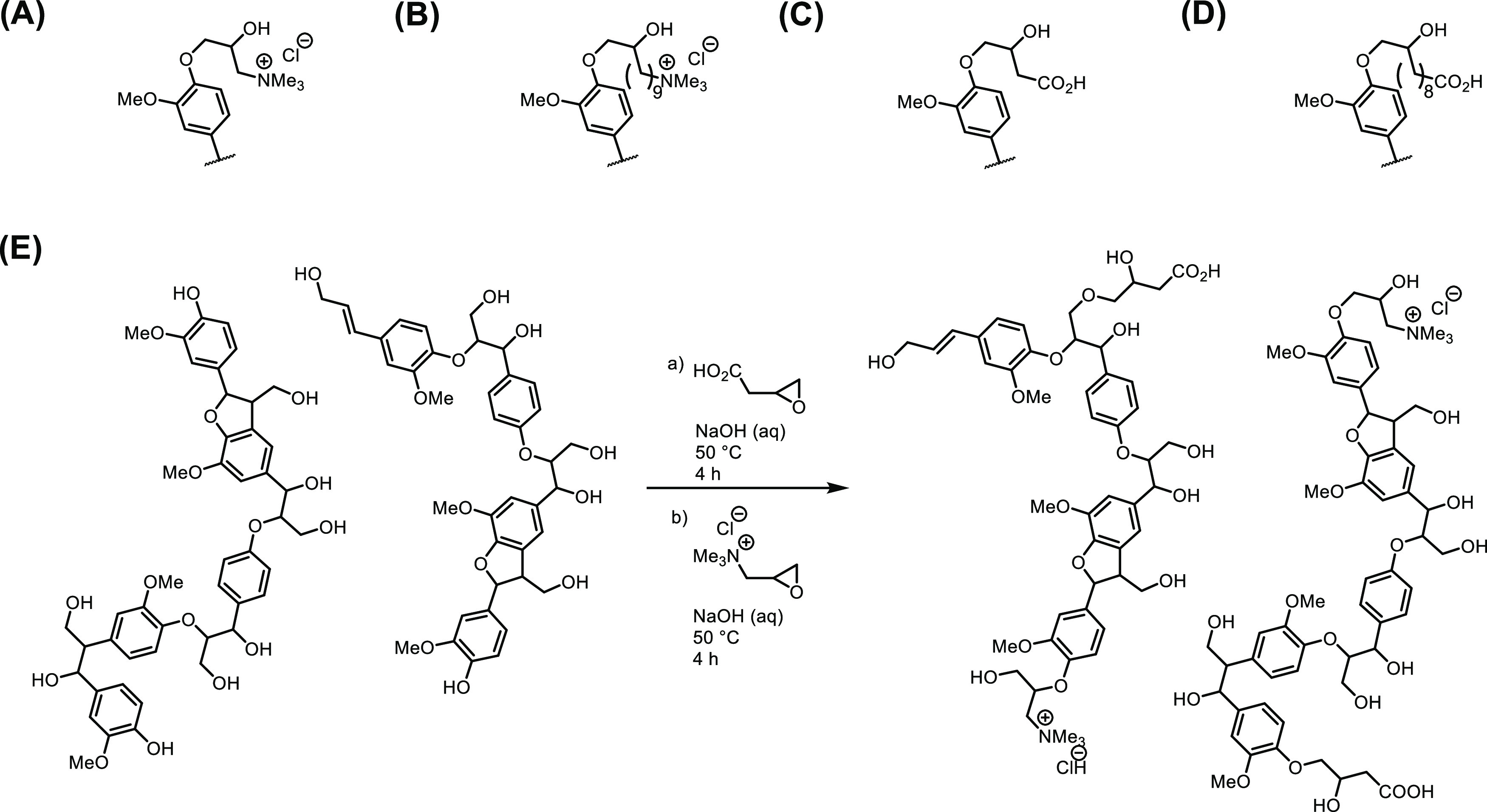
Functionalized OSL-derivatives Carrying Either (A,B) Permanent Positive Charge, (C,D) Inducible Negative Charge, or (E) Inducible Zwitterionic Character While Maintaining Overall OH-group Content

Approx. 5 mg of the small molecule, that is, the synthesized functionals shown in Scheme 1, were dissolved in 600 μL of CDCl_3_, and the solution was transferred into 5 mm NMR tubes. The spectra were acquired on a Bruker 300 MHz spectrometer or on a Bruker 400 MHz spectrometer using 64 scans at 20 °C within the standard zg pulse sequence. NMR data were processed with MestreNova (Version 8.1.1, Mestrelab Research).

### ^1^H NMR Analysis for Lignins

An accurately weighed amount of lignin (about 30 mg) was dissolved in 500 μL of DMSO-*d*_6_. A standard solution (100 μL) of 2,3,4,5,6-pentafluorobenzaldehyde in deuterated dimethyl sulfoxide (DMSO-*d*_6_) was then added, and the mixture was transferred into 5 mm NMR tubes. The spectra were acquired on a Bruker 300 MHz spectrometer or on a Bruker 400 MHz spectrometer using 64 scans at 20 °C within the standard zg pulse sequence. NMR data were processed with MestreNova (Version 8.1.1, Mestrelab Research).

### FT-IR Analysis

Fourier transform infrared (FT-IR) spectra were measured on a Perkin Elmer Spectrum 100 FTIR spectrometer operated with Spectrum software (version 2.45). The spectra were acquired in the form of potassium bromide pellets as the average of 32 scans between 450 and 4000 cm^–1^ with a resolution of 4 cm^–1^.

### Gel Permeation Chromatography Analyses

#### Method A

Approx. 3 mg of lignin or lignin derivative was dissolved in 1 mL of DMSO containing 0.1% lithium chloride. A Shimadzu instrument was used consisting of a controller unit (CBM-20A), a pumping unit (LC 20AT), a degasser (DGU-20A3), a column oven (CTO-20AC), a diode array detector (SPD-M20A), and a refractive index detector (RID-10A)), and controlled by Shimadzu LabSolutions (Version 5.42 SP3). A single analytical PLgel 5 μm MiniMIX-C column (Agilent, 250 × 4.6 mm) was used, being eluted at 70 °C with DMSO containing 0.1% lithium chloride. The run time at 0.25 mL min^–1^ flow rate was 20 min. Molecular weights were calculated from a linear calibration constructed with poly(styrene sulfonic acid) polymers (4.3–2600 kDa) in acid form and dimeric lignin models. Analyses were run in duplicate.

#### Method B

For GPC, approx. 5 mg of lignin or lignin derivative were acetobrominated^40^ and redissolved in tetrahydrofuran (THF) (3 mg/mL). Analysis was performed as detailed before,^41^ omitting the determination of correction factors. Analyses were performed using the Shimadzu hardware components described in Method A, employing three analytical GPC columns (each 7.5 × 30 mm) in series for analyses: Agilent PLgel 5 μm 10000 Å, followed by Agilent PLgel 5 μm 1000 Å, followed by an Agilent PLgel 5 μm 500 Å. High-performance liquid chromatography (HPLC)-grade THF (Chromasolv, Sigma-Aldrich) was used as eluent (0.75 mL min^–1^ for 70 min at 40 °C column temperature). Standard calibration was performed with polystyrene standards (Sigma-Aldrich, MW range 0.162–5000 kDa). Analyses were run in duplicate.

### Liquid Chromatography–Mass Spectrometry Analyses

Residual contents of potentially carcinogenic C_3_–NMe_3_Cl-functional in washing solutions as well as of 1% (m/m) suspensions of functionalized lignin, prepared in distilled water and filtered prior to injection, were analyzed by high-pressure liquid chromatography coupled to mass spectrometry (HPLC–MS) on the basis of suitable calibrations. Aliquots (1 mL) were taken and passed through a 0.45 μm syringe filter, before 20 μL was injected into the Shimadzu LC–MS system consisting of a pumping unit (LC 20A/B) with an in-built system controller (CBM-20Alite), a degasser (DGU-20A3), a diode array detector (SPD-M20A), and a final mass spectrometer with electrospray ionization (LCMS-2010EV) controlled by Shimadzu LCMSsolution (Version 5.42 SP3). A YMC-Pack Polymer C18 column (250 × 4.6 mm, 6 μm particle size) was eluted with acetonitrile-water [95/5 (v/v)], each containing 0.1% (v/v) formic acid. The run time at 0.5 mL min^–1^ flow rate was 30 min.

## Results and Discussion

### OSL-based Polyethers Carrying Permanently Charged or/and Chargeable Motifs

With the objective of developing very simple and cost-effective chemistries for lignin upgrade, we focused our attention on the control and selective modification of their phenolic groups, omitting as much as possible a reduction of the overall amount of hydroxyl groups in order to not unnecessarily diminish inherent bulk polarities. This precondition led to the choice of a general approach for adding “functionals” via formation of chemically stable alkyl aryl ether bonds, by anionic epoxide opening. The epoxide itself is linked to the “functional”, that is, the actually surface-active and/or surface-changing functional motif either by simple alkyl chains, or “directly” in case of polymeric “functionals”.

**Figure 2:**
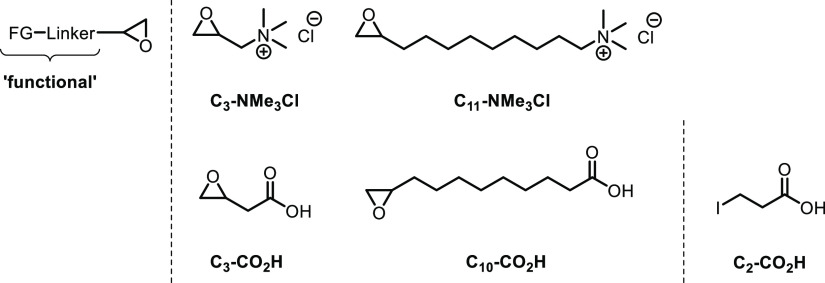
Epoxy-terminated monomeric “functionals” used for OSL functionalization. 3-Iodopropionic acid is used for generating a control species (compare Table 1).

Anionic opening of the terminal epoxides was achieved by phenolates generated at the lignin backbone upon treatment of OSLs with stoichiometric amounts of 1.0 M aqueous sodium hydroxide; this approach proved to be useful in our hands, also in unrelated, but in parallel pursued studies using glycidyl-terminated actives for functionalizations of a softwood kraft lignin (SKL),^42,43^ so that the literature-known alternative approach via cyclic carbonate-carrying functionals^44,45^ was dropped in light of the simplicity requirement. Figure 2 lists the monomeric “functionals” attached to the two different OSLs; Scheme 1 shows a representative reaction and realized structures.

Activation of the phenolic OH-groups along the lignin backbones was achieved by equilibrating the lignin with an amount of hydroxide ions that corresponded to the combined amount of phenolic OH-groups and carboxylic acid groups as determined by well-established quantitative ^31^P NMR after phosphitylation using 2-chloro-4,4,5,5-tetramethyl-1,3,2-dioxophospholane (2-Cl-TMDP);^38^ they were found to be 2.4 ± 0.3 and 2.6 ± 0.1 mmol per gram WS-OSL and CS-OSL, respectively (Table 1, entries 1 and 12). Blank reactions revealed that both WS-OSL and CS-OSL do not undergo major structural changes under these activation conditions (Table 1, entries 2 and 13).

**Table 1:** WS-OSL and CS-OSL Chemically Functionalized Using Monomeric Glycidyl-terminated Functionals

entry	lignin	“functional” (equiv)^a^	mass return [%] (reaction scale)	OH_arom_ + COOH [mmol/g]^b^	Mn [Da]^c^
1	WS-OSL			2.4 ± 0.3 (1.92 + 0.47)^d^	1300(1000^e^)
2		blank	85 (0.5 g)	2.6 ± 0.1 (1.95 + 0.62)^d^	1200 (930^e^)
3		C_3_–NMe_3_Cl (1.1)	80 (1.0 g)	1.7 ± 0.3 (89%)	1000^f^
4		C_3_–NMe_3_Cl (2.0)	78 (2.0 g)	1.6 ± 0.3 (84%)	n.d.
5		C_3_–NMe_3_Cl (1.0)	85 (6.0 g)	1.2 ± 0.3 (63%)	800^f^
6		C_9_–NMe_3_Cl (2.0)	87 (0.5 g)	0.9 ± 0.3 (47%)^f^	1400^f^
7		C_3_– CO_2_H (1.0)	90 (1.0 g)	0.7 ± 0.3 (36%)	1800
8		C_3_–CO_2_H (1.2)	87 (6.0 g)	1.0 ± 0.3 (52%)	1700
9		C_2_– CO_2_H (2.0)	91 (1.0 g)	0.7 ± 0.3 (36%)	1800
10		C_8_– CO_2_H (2.0)	100 (1.0 g)	<0.5 ± 0.3 (26%)^f^	1700
11		C_3_– CO_2_H (0.5) + C_3_–NMe_3_Cl (0.5) (two steps)	49 (1.0 g) (two steps)	(0.7 + 0.6) ± 0.3 (68% overall)	1600^f^
12	CS-OSL			2.6 ± 0.1 (2.38 + 0.23)^d^	1200 (1100^e^)
13		blank	64 (0.5 g)	2.3 ± 0.1 (2.04 + 0.25)^d^	1000 (900^e^)
14		C_3_–NMe_3_Cl (1.2)	22 (12 g)	1.6 ± 0.3 (68%)	500^e^^,^^f^
15		C_3_– CO_2_H (1.2)	92 (12 g)	0.4 ± 0.3 (17%)	1300^f^

a Acronyms as defined in Scheme 1; equivalents with respect to amount of activated phenolic OH-groups.

b As determined by quantitative ^31^P NMR; in case of functionalized lignins, numbers represent CONSUMED amount; % values in brackets indicate % of consumed phenolic OH-groups.

c DMSO-based single-column GPC-protocol (Method A), if not indicated otherwise.

d Accumulated amount of acidic OH-groups (phenolic + carboxylic).

e THF-based three-column GPC-protocol (Method B).

f Sample not fully soluble under any analysis condition.

In a first set of experiments, the so-generated OSL polyphenolates were reacted with “monomeric functionals” in order to introduce either a permanent positive charge (Table 1, entries 3–6, 14, Scheme 1A,B), an introducible negative charge (Table 1, entries 7–10, 15, Scheme 1C,D), or an introducible zwitterionic character (Table 1, entry 11; Scheme 1E).

**Figure 3:**
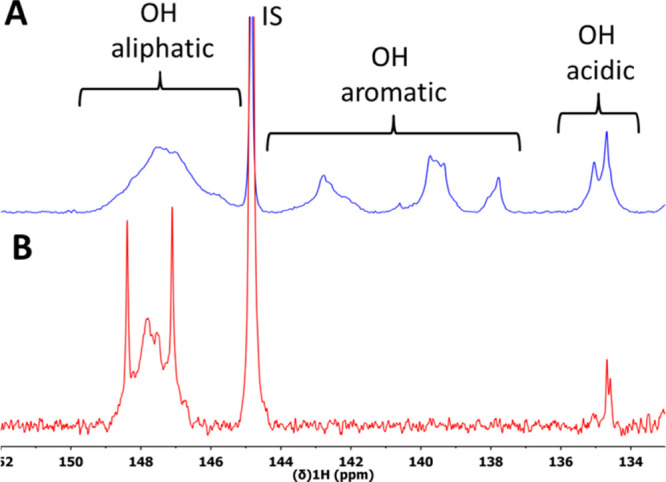
^31^P NMR spectra of phosphitylated WS-OSL (A) before and (B) after functionalization with C_3_–NMe_3_Cl (Table 1, entry 3); IS = internal standard (cholesterol).

The generation of an ammonium group-carrying and thus permanently positively charged lignin has been achieved using glycidyltrimethylammonium chloride, C_3_–NMe_3_Cl.^46,47^ In case of WS-OSL, functionalization proceeded smoothly with acceptable mass returns. FT-IR confirmed functionalization with characteristic bands in the fingerprint region at 978 and 918 cm^–1^ and an augmented signal for C–H-stretching at 2940 cm^–1^. ^1^H NMR spectroscopy indicated the presence of the ammonium functionality via a characteristic signal for the protons of the *N*-bound methyl groups. The loading of the functionalized lignin derivative was almost quantitative with approx. 1.7 mmol/g as determined by comparative quantitative ^31^P NMR analysis,^17,36,37^ corresponding to a consumption of approx. 89% of phenolic OH-groups. Figure 3 shows the ^31^P NMR spectra of this sample in comparison to the parent WS-OSL.

Increasing the amount of the ammonium “functional” did not lead to an increased technical loading in isolated material; this result hints at the fact that a technical loading of 1.6–1.7 mmol/g represents the maximum achievable loading.^42,43^ An intense coloring of the supernatants of the first isolation and the two subsequent washing cycles suggests that the drastically increased solubility of the “perfunctionalized” material also in acidified water (pH 2) leads to a loss of smaller functionalized lignin oligomers. An alternative work-up employing a dialysis protocol as proposed elsewhere for a kraft lignin functionalization has not been used in light of potential industrial boundary conditions.^46^ A LC–MS analysis of the supernatant of the last washing cycle and of the liquid phase of a 1% (m/m) suspension of redispersed functionalized lignin indicated a combined concentration of residual starting epoxide and quenching product 2,3-dihydroxy-*N*,*N*,*N*-trimethylpropan-1-aminium chloride of 10–100 ppm. As a category 1B carcinogenic compound, a maximum concentration of 1000 ppm (lower reactivity) to 100 ppm (higher reactivity) is allowed as the exposure limit for glycidyltrimethylammonium chloride, C_3_–NMe_3_Cl; the realized derivatized lignin sample thus complied with current exposure limits for potentially carcinogenic compounds of higher reactivity.

In a nonlinear upscaling experiment using unchanged conditions in terms of reaction time and temperature, the tuning and scaling possibilities were demonstrated: a less loaded derivative was produced using 0.5 equivalents of C_3_–NMe_3_Cl with respect to the amount of activated phenolic OH-groups. Quantitative ^31^P NMR analysis confirmed the envisaged approx. 50% of loading.

The hydrophobicity characteristics of ammonium-functionalized lignins can be tuned by the choice of the length of alkyl linker between the lignin backbone and functional group. Therefore, WS-OSL was functionalized with a permanent positive charge using *N*,*N*,*N*-trimethyl-9-(oxiran-2-yl)nonan-1-aminium chloride (C_9_–NMe_3_Cl), freshly synthesized in two steps adopting the protocols known from the literature, starting from 10-undecenyl chloride via epoxidation^33^ followed by quaternization of trimethylamine.^34^ The C_9_–NMe_3_-carrying lignin derivative was isolated in 87% mass return (Table 1, entry 6) and identified by ^1^H NMR and FT-IR spectroscopy. When compared with a short-linker homologue (C_3_–NMe_3_) of comparable loading (Table 1, entry 5), the longer linker led to a significant reduction in solubility and a clear hydrophobidization of surfaces.^48,49^

Introduction of a carboxylic acid functionality, and thus an inducible negative charge, was identified as promising derivatization as well. In order to achieve this without a net consumption in OH-groups, 2-(oxiran-2-yl)acetic acid, C_3_–CO_2_H, known from the literature was freshly synthesized^33^ and immediately reacted with lignin activated by sodium hydroxide. In order to prevent a reprotonation of the activated phenolics by C_3_–CO_2_H in acid form, this functional was actually added in the form of its sodium salt. Mass returns were nearly quantitative and slightly higher than for the ammonium-functionalized derivatives (Table 1, entries 7 and 8), thanks to the now more favorable p*K*_a_ of the novel lignin derivative. The carboxylic function was identified by ^1^H NMR spectroscopy peak at δ(^1^H) = 12.26 ppm and by FT-IR spectroscopy by an additional shoulder at 1765 cm^–1^. Technical loadings were determined once more on the basis of consumption of phenolic OH-groups. Identified loadings of 0.9 mmol/g were generally lower than in case of the ammonium derivatives. Efforts carried out in the presence of an excess of C_3_–CO_2_H did not yield significantly higher loading factors. The reason for this might lie in a charge repulsion effect. GPC analysis of C_3_–CO_2_H-functionalized lignin derivatives produced slightly higher number-average molecular weights in comparison to non-derivatized starting material. A nonlinear scale up of the reaction to 6 g resulted in an increase of the loading factor and in a nearly quantitative mass return (Table 1, entries 7 and 8), thus demonstrating again the tuning potential of the functionalization protocol.

**Figure 4:**
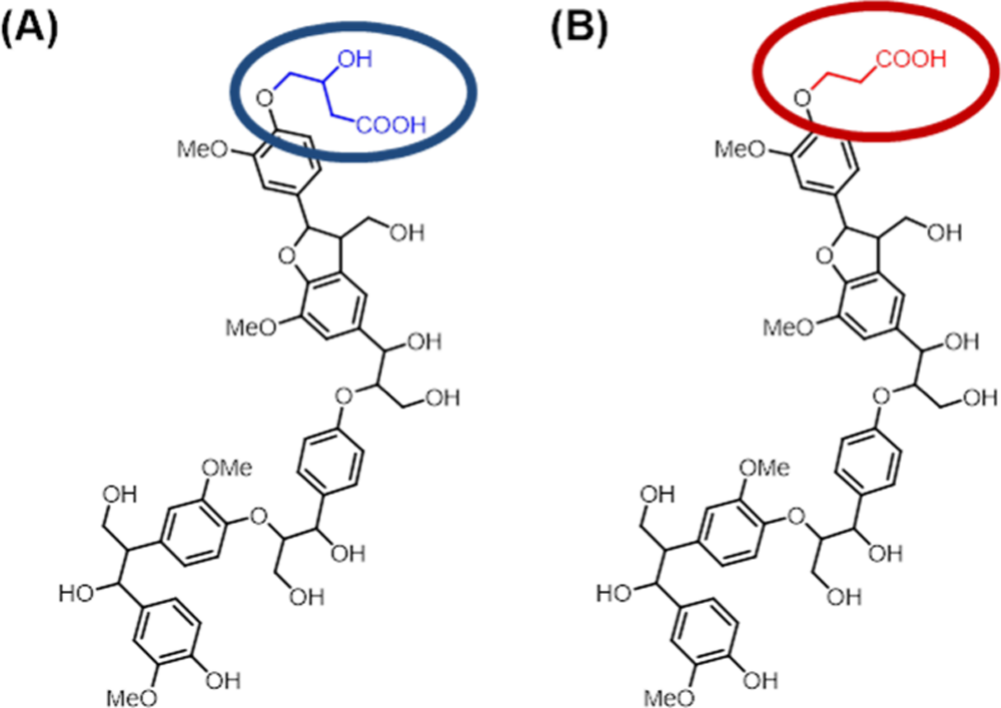
Structural differences between WS-OSL functionalized with (A) C_3_–CO_2_H and (B) C_2_–CO_2_H (Table 1, entries 7 and 9, respectively).

To highlight the benefit of the ’OH-group neutral’ functionalization, WS-OSL was functionalized with 3-iodopropionic acid (C_2_–CO_2_H) (Figure 4). The corresponding derivative, exhibiting a technical loading of 0.6 mmol/g (Table 1, entry 9) and a reduced overall OH content, was found to exhibit a significantly lower solubility, causing a turbidity of the solution at the concentration necessary for the intended applications.^50^ Also, C_2_–CO_2_H-functionalized WS-OSL does not show polymerization.

The acid function was also bonded to the WS-OSL backbone using the significantly longer linker epoxy-terminated C_8_–CO_2_H (Figure 2, Scheme 1D). C_8_–CO_2_H was obtained upon oxone-mediated epoxidation of undecylenic acid.^33^ A maximum loading of only approx. 26% was obtained. This is due most probably to a combination of the aforementioned charge repulsion in combination with the sluggish long linker. The C_8_–CO_2_H WS-OSL-derivative underperformed in comparison with a comparably loaded C_3_–CO_2_H homologue because of altered solubility even in alkaline media.^50^

In order to demonstrate the general feasibility and flexibility of the proposed functionalization protocol, a zwitterionic WS-OSL derivative a was generated using C_3_–Me_3_Cl- and C_3_–CO_2_H-functionals in a sequential functionalization process (Table 1, entry 11; Scheme 1E). FT-IR analysis confirmed the presence of both functional motifs by the appearance of the respective characteristic bands. As expected, the solubility characteristics are different compared to those of the homogeneously functionalized counterparts.^48−50^

The general applicability of the present functionalization protocol to OSLs of a different botanical origin was demonstrated by CS-OSL functionalization. An ammonium-functionalized and an acid-carrying derivative were realized using the same approach as for WS-OSL described above. Most interestingly, loadings were inferior for CS-OSL under the otherwise unchanged conditions possibly due to the noticeably lower solubility of CS-OSL, even in activated state, with respect to WS-OSL, causing the reaction to proceed less smoothly (Table 1, entries 12–15). As in case of WS-OSL, ammonium functionalization resulted in higher loading factors than carboxylate functionalization using identical linkers and connectivities. In application trials, derivatized CS-OSL performed likewise to derivatized WS-OSL of similar absolute functional group content per gram solid material.^48−50^

Technical loadings were determined in terms of consumed phenolic OH-groups because these groups represent the more reactive centers within the lignin backbone under the chosen reaction conditions, as demonstrated in other studies using a SKL.^42,43^ The structurally very different OSLs used in this study were found not to react as cleanly as the kraft lignin: analytical data confirm that functionalization occurs for the biggest part via the phenolic OH-groups, but involvement of some aliphatic groups cannot be excluded. Importantly, functionalization of the aliphatic OH-groups does not interfere with the overall aim to generate functionalized lignins without a net loss in OH-groups. The error that this “side reaction” causes in the determination of the technical loading was found to be of maximum 10% across monomeric functionals. Furthermore, epoxide opening is less regioselective for the chosen functionals in comparison to other epoxides studied for SKL functionalization,^42,43^ as indicated by the different sharper signals in the region of the phosphitylated aliphatic OH-groups in Figure 3B. Detailed studies have not been performed regarding this aspect.

### OSL-containing PDMS- and PEG-copolymers

For other types of homecare and personal care applications, substances comprising polyethylene glycol (PEG) and PDMS moieties are needed. In the frame of this study, such substances were envisaged to be copolymers generated on the basis of the two OSLs as part of smaller, eventually cross-linked polymer networks.

**Figure 5:**
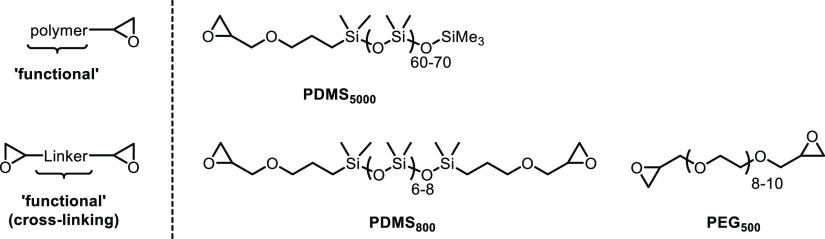
Epoxy-terminated mono- and bi-functional oligomeric and polymeric PEG and PDMS “functionals” used for OSL functionalization.

WS-OSL and CS-OSL were “copolymerized” with PEG and PDMS polymers/oligomers (Figure 5) under concomitant ether formation via the approach used in the addition of the above-discussed monomeric functionals. Results are summarized in Table 2.

**Table 2:** WS-OSL, CS-OSL, and Functionalized WS-OSL, CS-OSL, Chemically Copolymerized with PEG- and PDMS-functionals

entry	lignin	“functional” (equiv)^a^	emulsifier (% v/v)^b^	mass return [%](reaction scale)	OH_arom_ + COOH [mmol/g]^c^	Mn [Da]^d^ (PDI)
1	WS-OSL				2.4 ± 0.3 (1.92 + 0.47)^e^	1000 (4.1) (1300 (7.3)^e^)
2		blank		82 (0.5 g)	2.6 ± 0.1 (1.95 + 0.62)^e^	930 (8.2)
3		PDMS_5000_ (5.0)	FA-7EO (5)	117 (0.5 g)	n.d.^f^	1200 (>10)^g^
4		PDMS_800_ (5.0)	FA-7EO (5)	129 (0.5 g)	0.5 ± 0.3	1300 (>10)^g^
5		PEG_500_ (0.1)		84 (0.5 g)	0.1 ± 0.3	1200 (5.8)
6		PEG_500_ (0.5)		95 (1.0 g)	0.3 ± 0.3	1800 (>10)
7		PEG_500_ (10)		88 (0.5 g)	1.4 ± 0.3	2200 (6.9)
8		C_3_–NMe_3_Cl + PEG_500_ (0.6 + 0.5, two steps)		65 (0.5 g) (two steps)	(0.6 + 0.3) ± 0.3	600 (5.0)^g^
9		C_3_–CO_2_H + PEG_500_ (two steps)		78 (0.5 g) (two steps)	(0.4 + 0.3) ± 0.3	2150 (>10)^g^^,^^h^
10	CS-OSL				2.6 ± 0.1 (2.38 + 0.23)^e^	1100 (4.2)
(1200 (3.7)^e^)						
11		blank		64 (0.5 g)	2.3 ± 0.1 (2.04 + 0.25)^e^	900 (10)
12		PDMS_5000_ (1.0)	FA-7EO (5)	72 (0.5 g)	0.4 ± 0.3^g^	1600 (6.8)^g^
13		PDMS_800_ (1.1)	FA-7EO (5)	92 (0.5 g)	1.0 ± 0.3^g^	1500 (4.7)^g^

a Acronyms as defined in Scheme 1; equivalents with respect to max amount of activatable phenolic groups as determined by quantitative ^31^P NMR.

b Lauryl heptaethoxylate.

c As determined by quantitative ^31^P NMR; in case of functionalized lignins, numbers represent CONSUMED amount.

d THF-based three-column GPC-protocol (Method B), if not stated otherwise; PDI = polydispersity index.

e Accumulated amount of acidic OH-groups (phenolic + carboxylic).

f Not delineable, as product soluble under analysis conditions.

g Isolated material not fully soluble under standard conditions for measurements.

h DMSO-based single-column GPC-protocol (Method A).

**Scheme 2:**
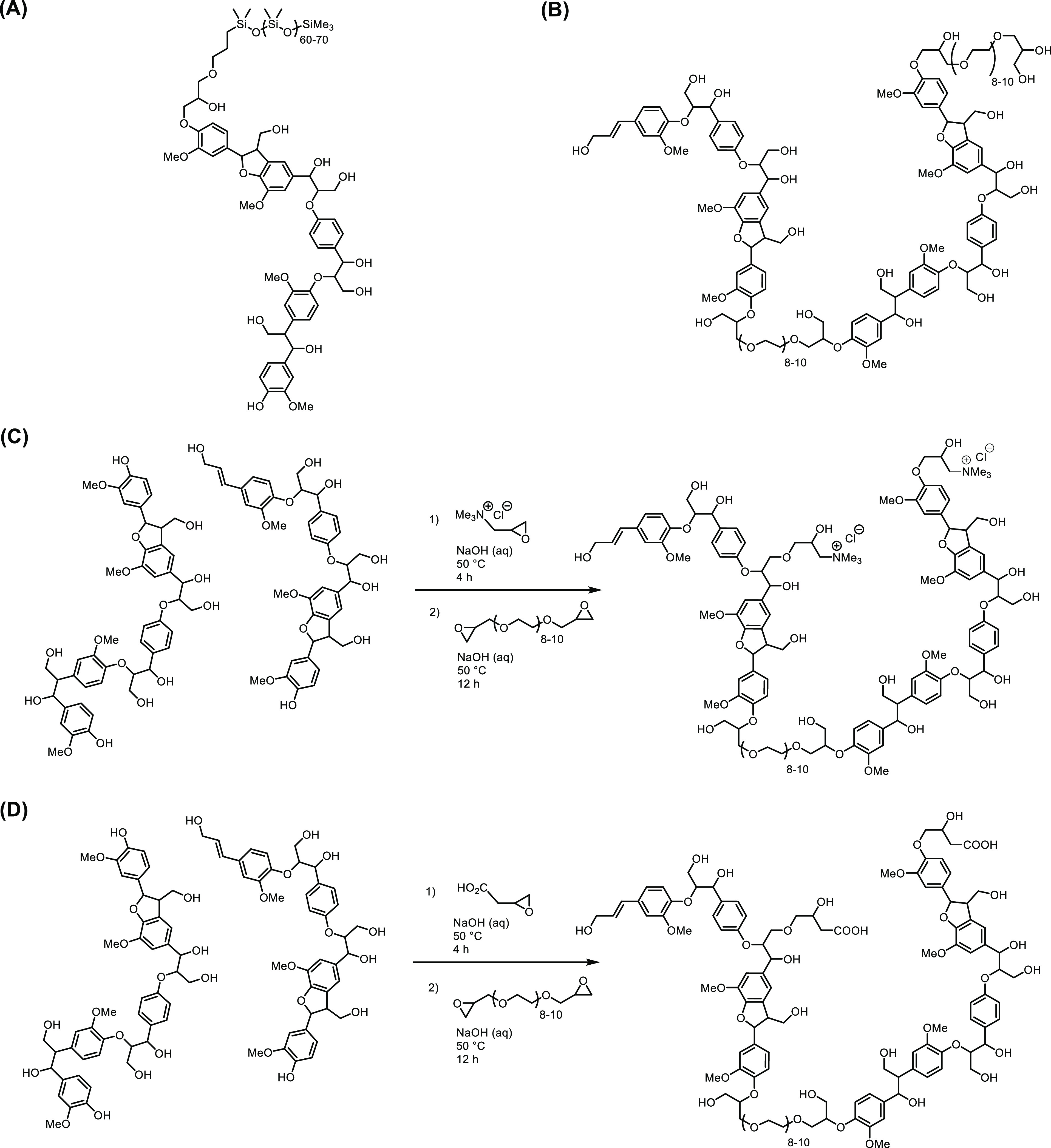
Schematic Exemplary Structures for Multiply Functionalized WS-OSLs as Described in Table 2, (A) Entry 3, (B) Entry 5 (as Reaction), (C) Entry 8 (as Reaction), and (D) Entry 9 (as Reaction)

PDMS-containing WS-OSL-based copolymers were generated using monoglycidyl-terminated functional PDMS_5000_ and diglycidyl-terminated PDMS_800_ (Scheme 2). Employing the monofunctional PDMS-functional lead to oligomeric lignin chains^31^ decorated with a limited number of tangling PDMS-polymer chains, hence de facto a PDMS-derivative containing a low weight-percentage of lignin (Table 2, entry 3). Isolable materials consisted of a dark-brown, soft solid and a light brown viscous oil. The oil, isolated in 78% with respect to the amount of the PDMS starting functional, turned out to be fully non-analyzable with our means; the brown solid was difficult to handle in any type of standard analysis. ^1^H NMR analysis of the soluble part, accounting for only roughly 40% of the sample, did indicate the presence of PDMS-based methyl groups next to lignin-typical signals.

**Figure 6:**
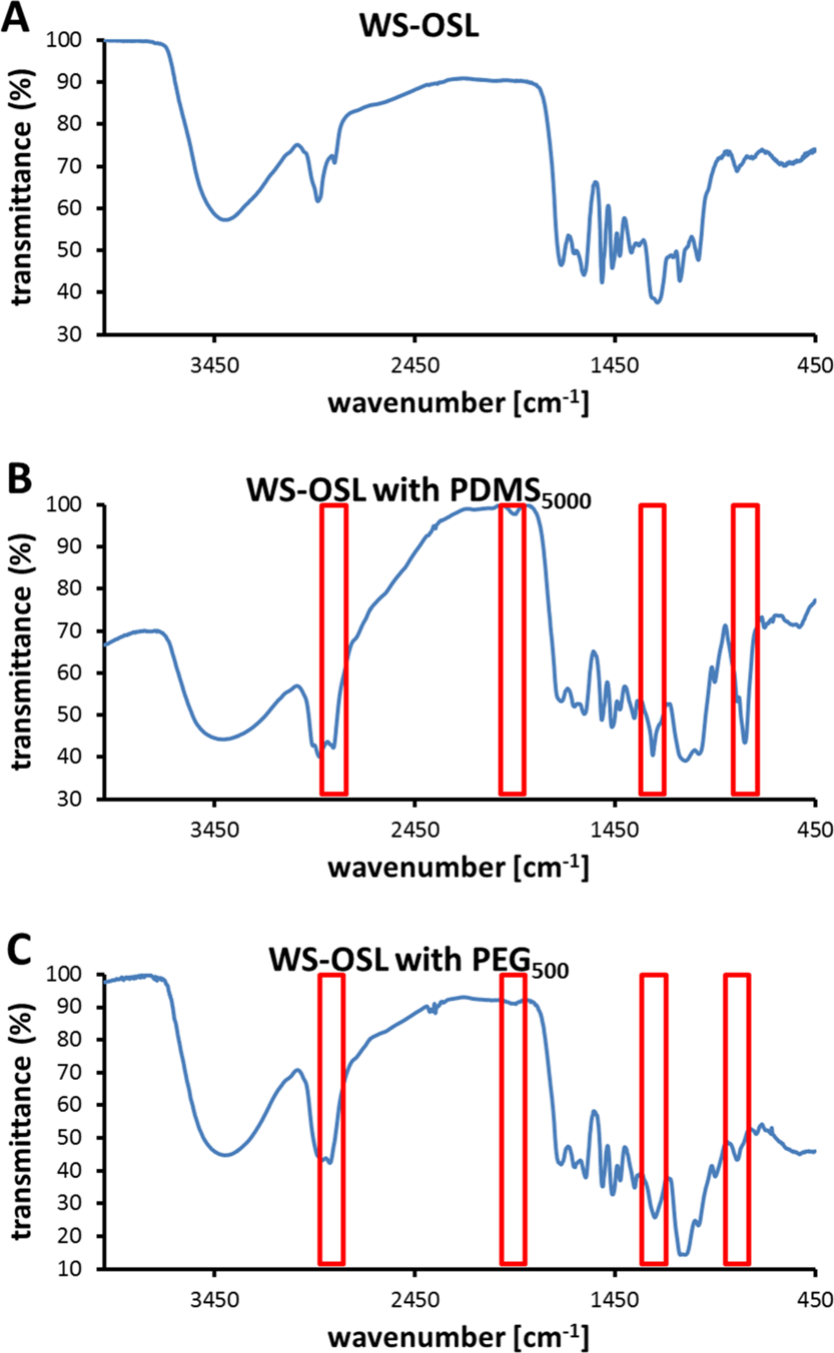
FT-IR analyses of (A) WS-OSL functionalized with (B) PDMS_5000_ (Table 2, entry 5) and (C) PEG_500_ (Table 2, entry 6).

FT-IR confirmed the presence of functional groups typical for the lignin and for the PDMS functional in this product (Figure 6A): very strong bands for CH-stretches are visible at 2926 and 2856 cm^–1^; a very strong deformed band peaking at 1260 cm^–1^ is indicating both typical lignin motifs and Si–CH_3_ groups. The fingerprint region, showing in the parent WS-OSL distinct bands representing C–O stretching of alkyl ethers at 1158 cm^–1^ and 1125 cm^–1^, respectively, is less resolved for the PDMS-lignin derivative; only the third band typical for these groups at 1031 cm^–1^ is clearly visible in both spectra. The first two are covered by a broad strong band peaking at 1100 cm^–1^, attributable to various Si–O–Si stretchings.

The introduced structural changes conferred to the lignin base structures a significantly altered molecular weight. Large polydispersities rendered it practically impossible to determine a meaningful technical loading in terms of consumed OH-groups; the phenolic OH group content as determined by quantitative ^31^P NMR can nevertheless per se be treated as a specific number suitable for structurally characterizing the novel lignin-containing copolymers.

GPC analysis of the soluble part of the sample revealed an increase of the mean average molecular weight by just 20% with respect to the starting material. A practically identical situation was encountered when CS-OSL was reacted with PDMS_5000_ under identical conditions Table 2, entry 12.

Production of crosslinked copolymeric PDMS-lignin derivatives by reacting WS-OSL with PDMS_800_ resulted in an oil and a brown paste as two primary products (Table 2, entry 4). As before, only the paste could be roughly analyzed under the standardized conditions and setups: for the parts soluble under the respective analysis conditions, an elevated Mn of 1300 Da was observed. Formal ^31^P NMR analyses suggested a characterizing loading number of 0.4 mmol/g. FT-IR-analysis confirmed the presence of both oligomeric building blocks as well, displaying the typical bands for lignin and PDMS as discussed in case of derivatization with PDMS_5000_. Most interestingly, CS-OSL seems to react significantly more smoothly with the smaller PDMS functional (Table 2, entry 13). As in case of the monofunctional building block, product analysis by FT-IR proved the presence of both block polymers. A higher technical loading of formal 1.2 mmol/g, technically corresponding to 50% consumed phenolic OH-groups, indicates that the soluble part of the pasty product represents an efficient mix of PDMS blocks with lignin oligomers. GPC analysis indicated a significant increase in Mn and thus supports the interpretation of the ^31^P NMR results.

As expected, lignin-PDMS copolymeric/co-oligomeric substances lead to a significant hydrophobization of hard surfaces.^48,49^

In the present study, various lignin-PEG_500_ ratios were reacted, such as to achieve various degrees of PEG-crosslinked WS-OSL (Table 2, entries 5–7): sub-stoichiometric amounts, that is, 0.1 and 0.5 equivalents of glycidyl-groups with respect to phenolic OH-groups per gram lignin, corresponding to approx. 5% (w/w) and 25% (w/w) of PEG with respect to lignin, respectively, led to true PEG-ylated lignins (Table 2, entries 5 and 6). An excess of PEG_500_, that is, 500% (w/w) to lignin, led to “lignified” PEG instead (Table 2, entry 7). Products were generally isolated with low mass returns due to the significantly enhanced solubility of the new species in water. Products were identified by the newly appearing peak for the protons of the OCH_2_-groups in the attached PEG chains at δ = 3.64 ppm. The FT-IR spectra indicated strong C–H-stretching at 2916 and 2874 cm^–1^; bands typical for the stretching and vibration of OCH_2_-groups were increased in intensity at around 1250 and 801 cm^–1^ (Figure 6B).

Permanently charged and chargeable PEG-ylated lignin copolymers shown in Scheme 2C,D were realized (Table 2, entries 8 and 9) using C_3_–NMe_3_Cl and C_3_–CO_2_H, respectively, in combination with PEG_500_. In two-step processes, the monomeric functionals were added first, aiming for a medium loading that would allow additional crosslinking via PEG_500_ in a second step using 25% (w/w) PEG with respect to lignin. ^1^H NMR and FT-IR analyses confirmed the presence of all functional groups in the isolated powders; ^31^P NMR confirms covalent binding via consumption of a total of approx. 47 and 37% of phenolic OH-groups for ammonium- and carboxyl-containing lignin-PEG co-oligomers, respectively.

GPC analysis of the soluble fractions reflect the dual nature of the products: mean average molecular weights lie in between those obtained for the derivatives functionalized with the two monomeric functionals and the PEG-linked lignins. Once generated, hybrid materials were tested in standard homecare formulations.^48,49^

Reaction of oligomeric and polymeric functionals were met with significantly more difficulties than the ones using the monomeric functionals. A less efficient consumption of the epoxides was expected and partly observed, as exemplarily indicated by the “pending” PEG moiety in the product shown in Scheme 2B. An estimation of how many of the aliphatic OH-groups react in case of the copolymer formation with the epoxides of the functionals has not been made for the reasons outlined above regarding the absoluteness of the ^31^P NMR in case of copolymeric structures.

### OSL Functionalized Using Enzymatic Catalysis

An alternative green approach to lignin functionalization can be performed by using oxidative enzymes. Polyphenol oxidases and LACs have been used in the past to catalyze lignin oxidation/depolymerization.^29,51,52^ In our effort, the enzymes were used as initiators of a radical reaction between enzyme-generated lignin phenoxy radicals^29,53^ and the active epoxide. In order to avoid as much as possible an uncontrolled following reaction of this radical with yet unreacted functionals, reactions were performed using rather high enzyme loadings, high dilutions, and a sequential addition of functionals. The first factor contributes to the formation of a high density of activated phenolic OH-groups. The high dilution of lignin and the sequential addition of reactants were meant to avoid intermolecular reactions. LAC-mediated reactions are summarized in Table 3.

**Table 3:** WS-OSL and CS-OSL Functionalized in form of Block Copolymers and Oligomers Using LAC (20 U per 100 mg Lignin)

entry	lignin	“functional” (equiv)^a^	emuls.^b^	mass return [%] (reaction scale)	technical loading [g/mmol]^c^	Mn [Da]^d^ (PDI)
1	WS-OSL				2.4 ± 0.3 (1.92 + 0.51)^e^	1000 (4.1)^f^470 (2.8)
2		blank		93 (0.5 g)	1.9 ± 0.3 (1.59 + 0.33)^e^	1100 (3.6)
3		PDMS_5000_ (1.0)	FA-7EO	80 (0.5 g)	0.2 ± 0.3^f^	1300 (9.0)^f^
4		PDMS_800_ (10)	FA-7EO	104 (0.5 g)	0.3 ± 0.3^f^	1600 (>10)^f^
5		PEG_500_ (0.1)		95 (0.5 g)	0.4 ± 0.3	1200 (5.2)
6		PEG_500_ (10)		76 (0.5 g)	0.8 ± 0.3	1700 (>10)
7	CS-OSL				2.6 ± 0.1 (2.38 + 0.23)^e^	1100 (4.2)^f^360 (3.9)
8		blank		91 (0.5 g)	2.4 ± 0.1 (2.08 + 0.26)^e^	1300 (4.5)
9		PDMS_5000_ (1.0)	FA-7EO	91 (0.5 g)	0.9 ± 0.3^f^	1500 (9.3)^f^
10		PDMS_800_ (10)	FA-7EO	103 (0.5 g)	1.5 ± 0.3^f^	1700 (>10)^f^
11		PEG_500_ (0.1)		98 (0.5 g)	0.4 ± 0.3	1200 (3.3)
12		PEG_500_ (10)		76 (0.5 g)	1.1 ± 0.3	1700 (6.1)

a Equivalents with respect to max amount of activatable phenolic groups as determined by quantitative ^31^P NMR.

b Lauryl alcohol ethoxylate displaying seven ethoxy groups.

c As determined by quantitative ^31^P NMR; in case of functionalized lignins, numbers represent CONSUMED amount.

d THF-based three-column GPC-protocol (Method A), if not stated otherwise; PDI = polydispersity index.

e Accumulated amount of acidic OH-groups (phenolic + carboxylic).

f Isolated material not fully soluble under standard conditions for measurements.

LAC treatment of WS-OSL and CS-OSL without addition of a functional led to an expectable slight polymerization of the OSLs under concomitant consumption of phenolic OH-groups (Table 3, entries 1 and 2, 7, and 8),^54,55^ as shown by GPC and quantitative ^31^P NMR spectroscopy.

When the PDMS-functionals, PDMS_5000_ and PDMS_800_ are added to the mix of WS-OSL and LAC in the presence of a nonionic surfactant, it leads to products similar in appearance and consistence to the corresponding products from the chemical transformations discussed above. Analyses of the products showed increased molecular weights and a consumption of phenolic OH-groups above the level obtained for the background polymerization, indicating that the PDMS-functionals were linked to the lignin backbone via the lignin phenolic end groups.

CS-OSL–PDMS copolymers were obtained under analogous reaction conditions. Also in this case, the reaction course yielded products (Table 3, entries 9 and 10) comparable to those obtained by chemical modification (Table 2, entries 12 and 13) in terms of product formation, formal technical loading, and achieved polymerization.

The same approach was used to obtain LAC-mediated PEG-ylation of WS-OSL using low weight percentages of PEG_500_. The products obtained showed comparable analyses data as the chemical counterparts discussed above (Table 3, entry 5 vs. Table 2, entries five and 6). At higher weight percentages of PEG_500_, the actual lignification of PEG_500_ was enzymatically realized (Table 3, entry 6). In this case, however, the chemical reaction was significantly more effective than the enzymatic one in the production of a clearly polymerized material (Table 2, entry 7). The trends observed in the production of PEG–WS-OSL hybrid materials were practically duplicated during the incubation of two different PEG_500_–CS-OSL mixes (Table 3, entries 11 and 12). Standard analyses indicate successful product formation and polymerization.

## Conclusions

A general methodology for selective functionalization of OSLs with small functional groups introducing permanent or inducible charges was developed. Wheat straw and corn stover organosolv lignin, WS-OSL and CS-OSL, were successfully functionalized with various degrees of technical loadings. The same strategy was found suitable for the synthesis of PEG and PDMS lignin copolymers of different sizes.

Alternatively, LAC as a radical reaction initiator was applied to generate ether-linked PDMS- and PEG-lignin copolymers. Overall, this LAC-mediated block-copolymerization of lignins turned out as a competitive process compared to the chemical functionalization of the OSLs under study and represents thus a very important finding within the sustainability aspect, also in light of potential applications.

## Supporting Information Available

^31^P NMR spectra of WS-OSL and OS-WSL starting lignins as well as ^31^P NMR spectra of realized derivatives (PDF)
