# Supplementary material for: Functionalized Organosolv Lignins Suitable for Modifications of Hard Surfaces
Source: ACS Sustain Chem Eng. 2020 Apr 20;8(20):7628–38. doi: 10.1021/acssuschemeng.0c00886 (PMC8016396; doi:10.1021/acssuschemeng.0c00886)

FUNCTIONALISED ORGANOSOLV LIGNINS SUITABLE FOR MODIFICATIONS OF HARD SURFACES

SUPPORTING INFORMATION

Paola Giannì[a], Heiko Lange[b,c,†,]*, Claudia Crestini[c,d,†,]*

a: Department of Chemical Sciences and Technologies, University of Rome 'Tor Vergata', Via della Ricerca Scientifica, 00133 Rome, Italy

b: Department of Pharmacy, University of Naples 'Federico II', Via Domenico Montesano 49, 80131 Naples, Italy

c: CSGI - Center for Colloid and Surface Science, Via della Lastruccia 3, 50019 Sesto Fiorentino, Italy

d; Department of Molecular Science and Nanosystems, University of Venice Ca' Foscari, Via Torino 155, 30170 Venice Mestre, Italy

†: Also affiliated with a) *via* NAST – Nanoscience & Nanotechnology & Innovative Instrumentation Center.

* Corresponding authors: heiko.lange@unina.it
claudia.crestini@unive.it

Table S1: Representative ^{31}P NMR spectra of realised functionalised **WS-OSL** and **WS-OSL**-based copolymer functionalised either chemically or *via* enzymatic catalysis as described in the manuscript.

WS-OSL (starting material)

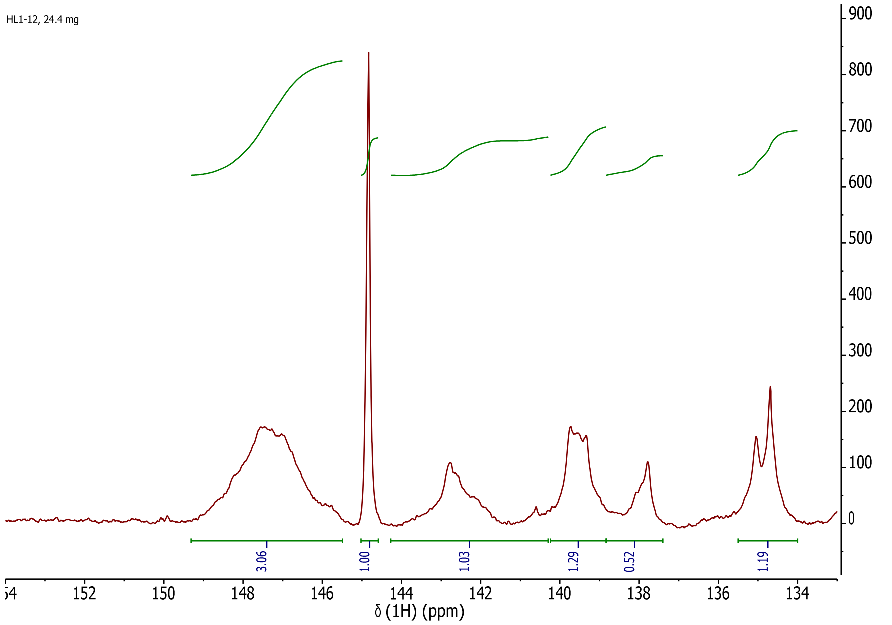

WS-OSL blank

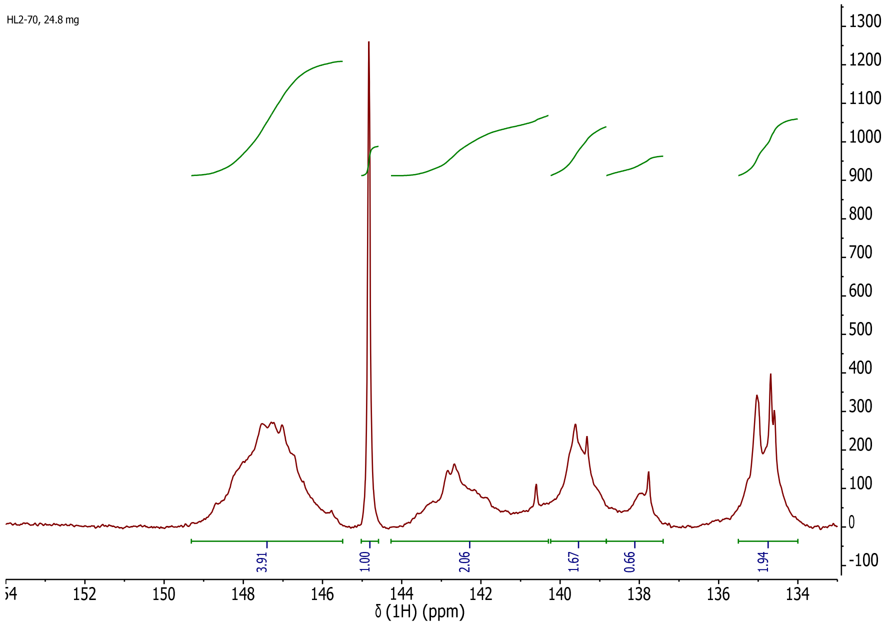

Table S1 (continued): Representative ^{31}P NMR spectra for realised functionalised **WS-OSL** and **WS-OSL**-based copolymer functionalised either chemically or *via* enzymatic catalysis as described in the manuscript.

WS-OSL + C_3-NMe_3Cl (2.0 eq.) [Table 1, entry 4]

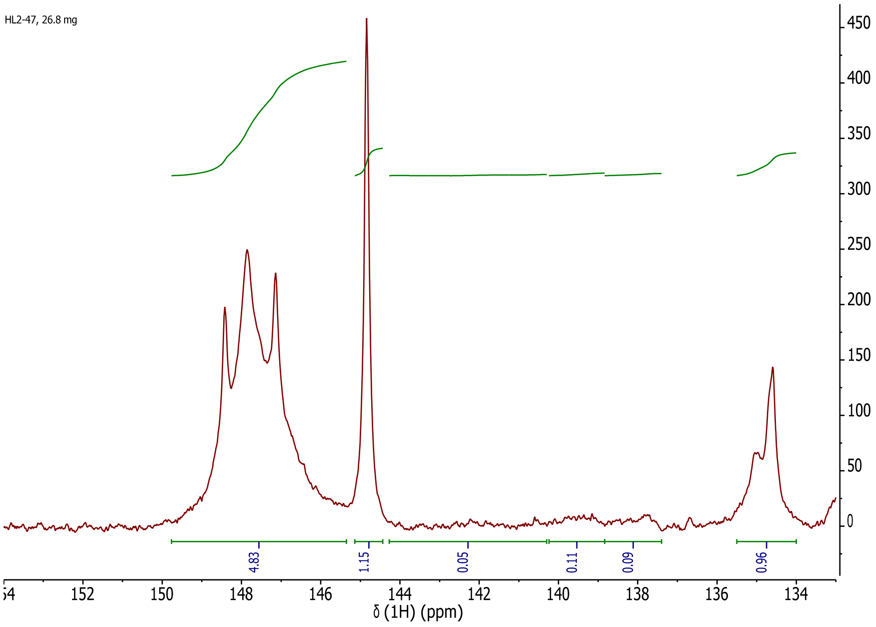

WS-OSL + C_3-NMe_3Cl (1.0 eq.) [Table 1, entry 5]

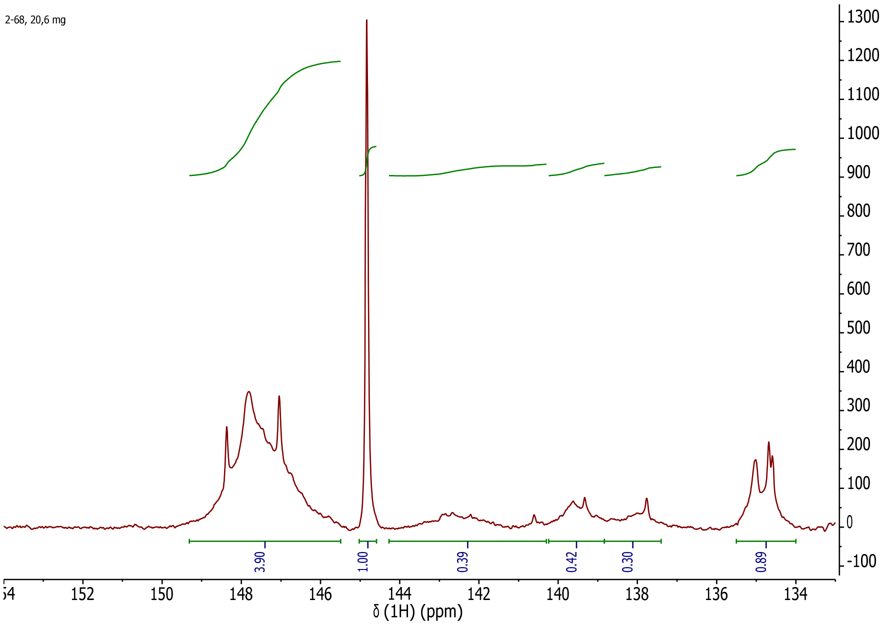

Table S1 (continued): Representative ^{31}P NMR spectra for realised functionalised **WS-OSL** and **WS-OSL**-based copolymer functionalised either chemically or *via* enzymatic catalysis as described in the manuscript.

WS-OSL + C_9-NMe_3Cl (2.0 eq.) [Table 1, entry 6]

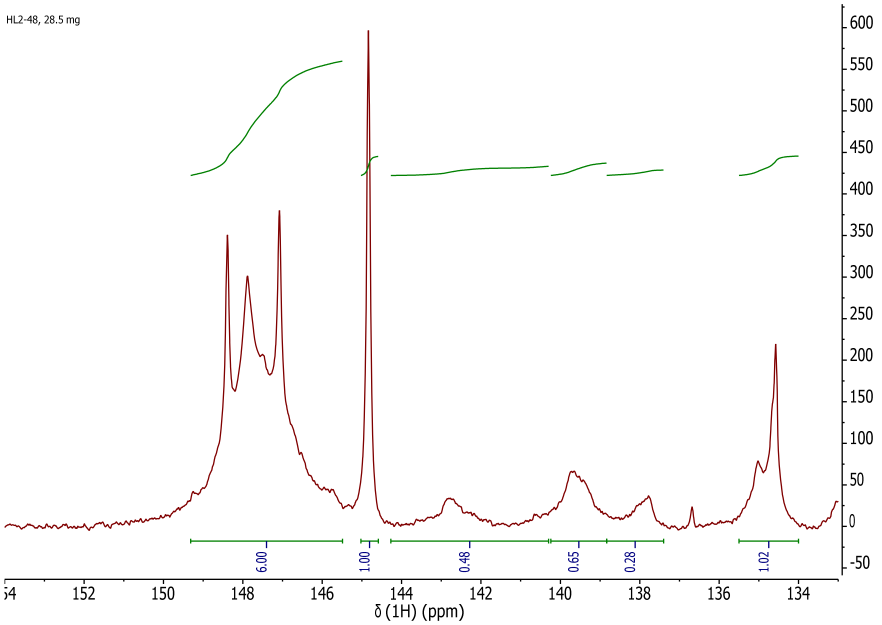

WS-OSL + C_3-CO_2H (1.0 eq.) [Table 1, entry 7]

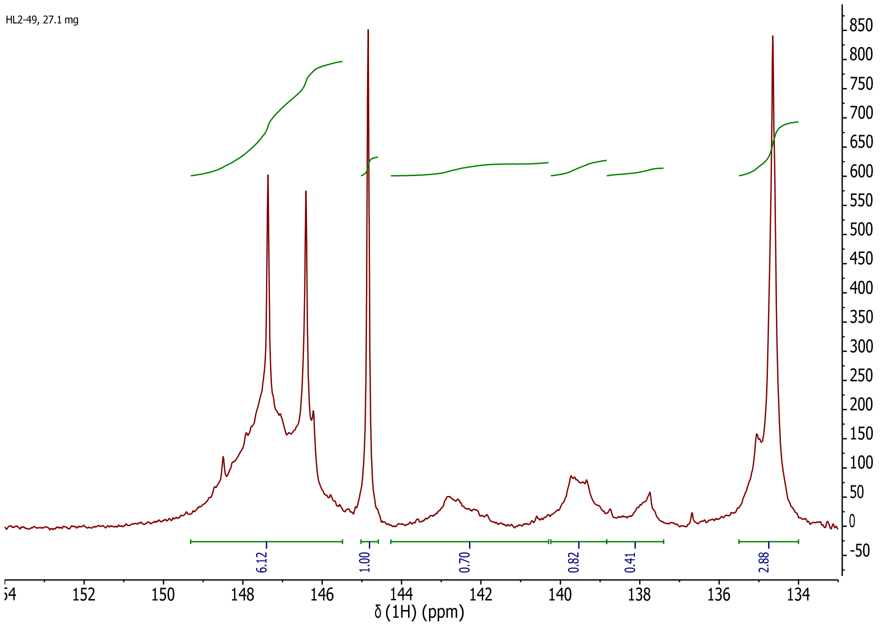

Table S1 (continued): Representative ^{31}P NMR spectra for realised functionalised **WS-OSL** and **WS-OSL**-based copolymer functionalised either chemically or *via* enzymatic catalysis as described in the manuscript.

WS-OSL + C_2-CO_2H (2.0 eq.) [Table 1, entry 9]

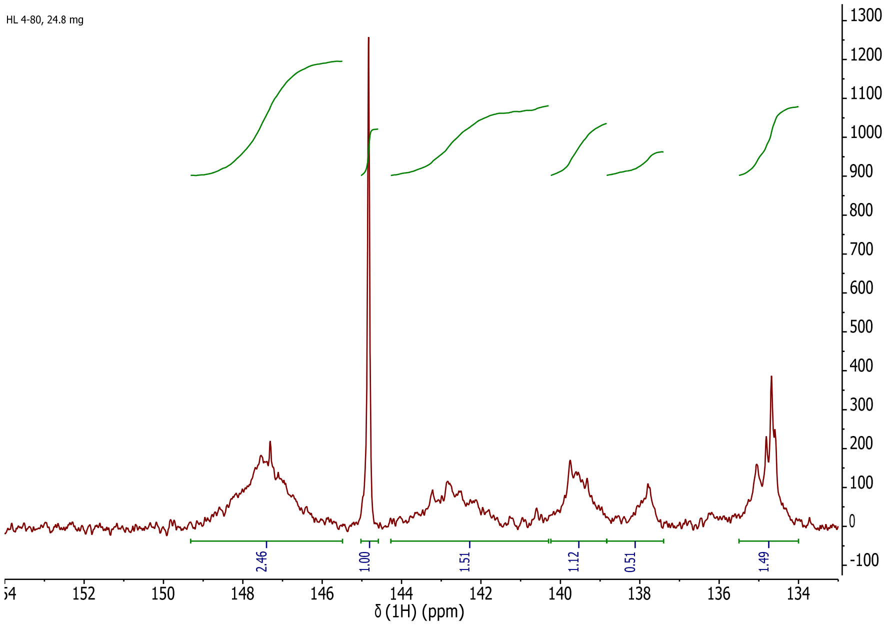

WS-OSL + C_8-CO_2H (2.0 eq.) [Table 1, entry 10]

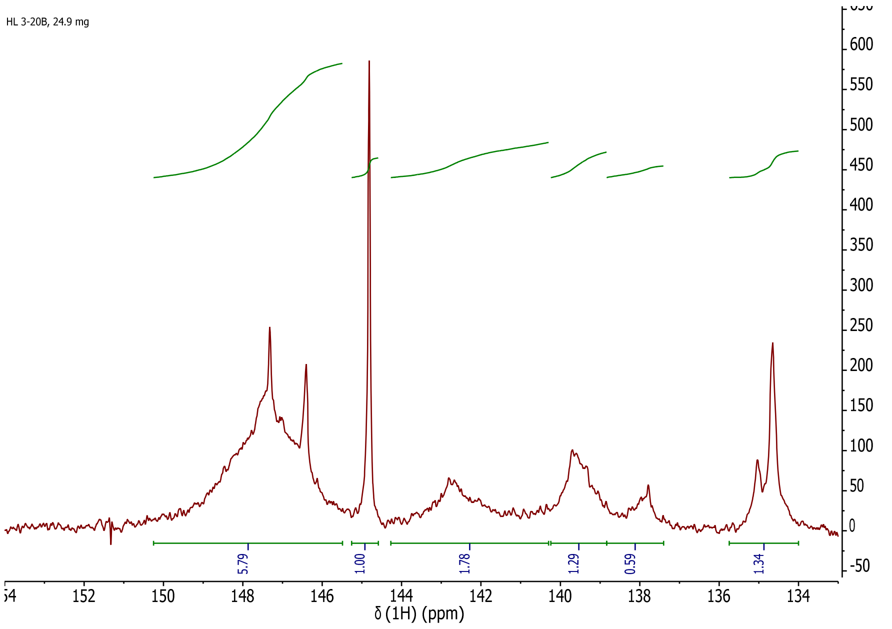

Table S1 (continued): Representative ^{31}P NMR spectra for realised functionalised **WS-OSL** and **WS-OSL**-based copolymer functionalised either chemically or *via* enzymatic catalysis as described in the manuscript.

WS-OSL + C_3-CO_2H (0.5 eq.) + C_3-NMe_3Cl (0.5 eq.) [Table 1, entry 11]

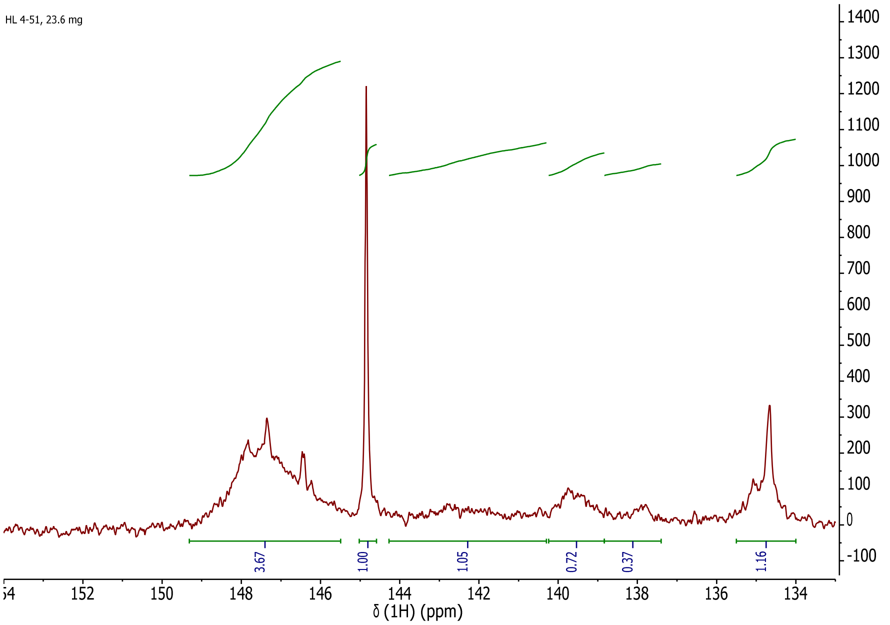

WS-OSL + $PDMS_{800}$ (5.0 eq.) [Table 2, entry 4]

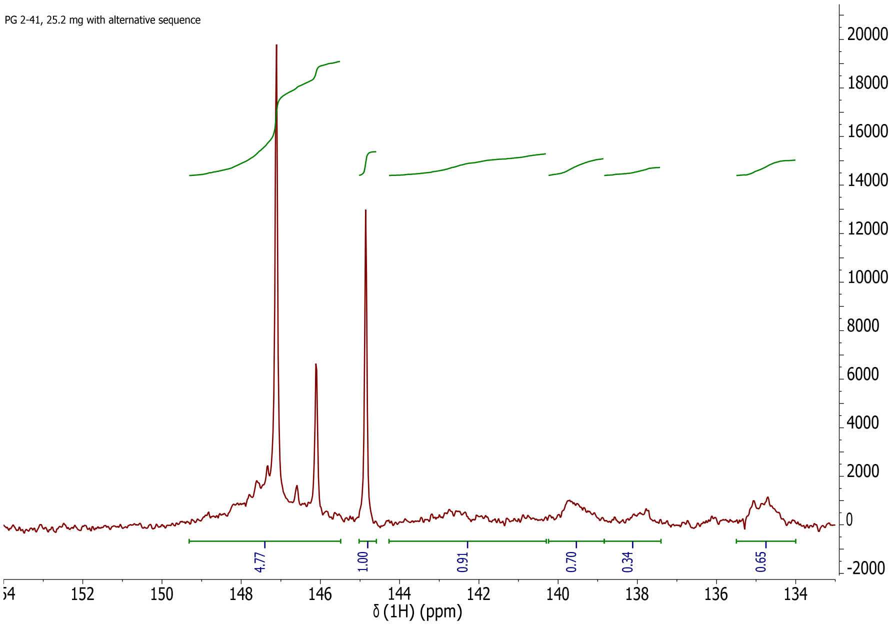

Table S1 (continued): Representative ^{31}P NMR spectra for realised functionalised **WS-OSL** and **WS-OSL**-based copolymer functionalised either chemically or *via* enzymatic catalysis as described in the manuscript.

WS-OSL + PEG_{500} (10 eq.) [Table 2, entry 7]

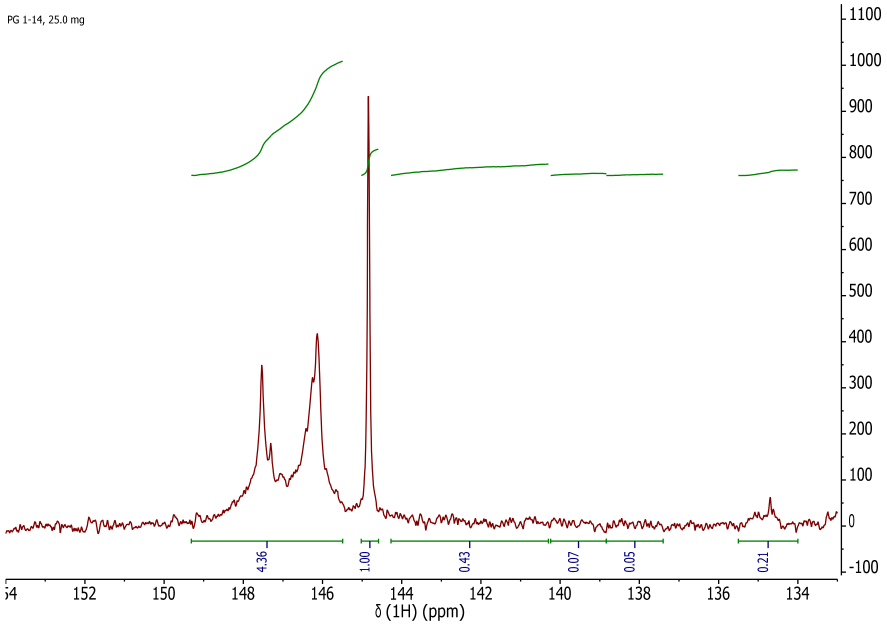

WS-OSL + C_3-NMe_3Cl (0.6 eq.) + PEG_{500} (0.5 eq.) [Table 2, entry 8]

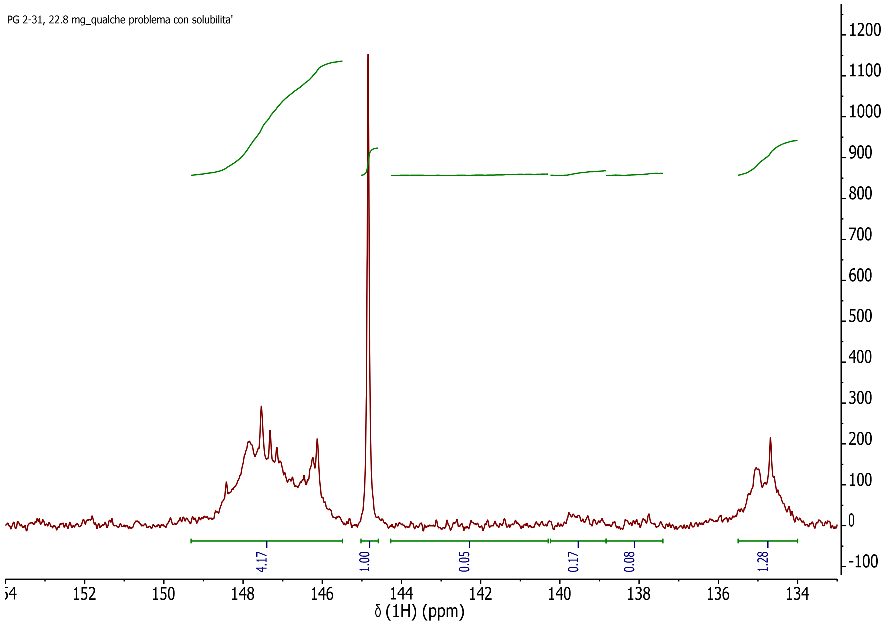

Table S1 (continued): Representative ^{31}P NMR spectra for realised functionalised **WS-OSL** and **WS-OSL**-based copolymer functionalised either chemically or *via* enzymatic catalysis as described in the manuscript.

WS-OSL + C_3-CO_2H (0.6 eq.) + PEG$_{500}$ (0.5 eq.) [Table 2, entry 9]

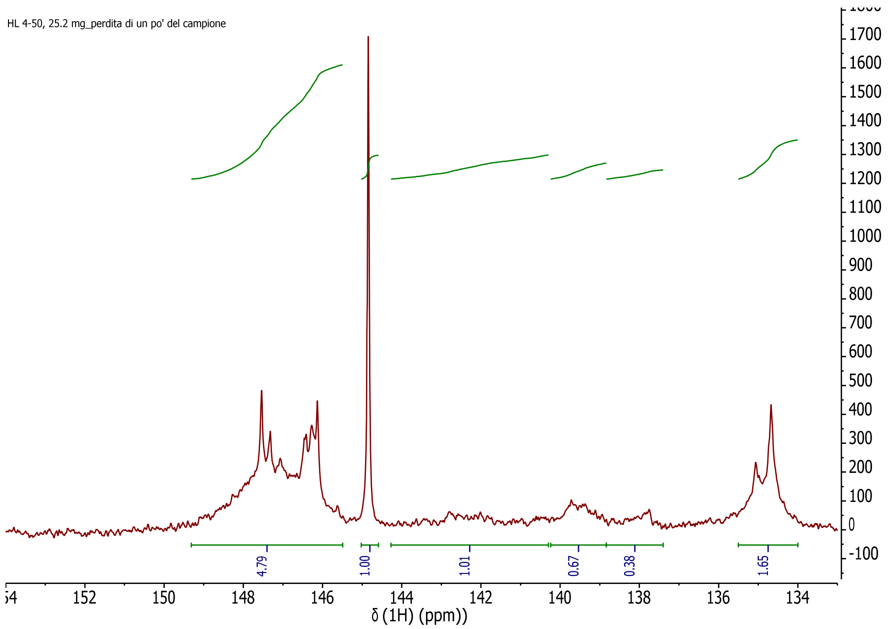

WS-OSL + PDMS$_{5000}$ (1.0 eq.) [Table 3, entry 3]

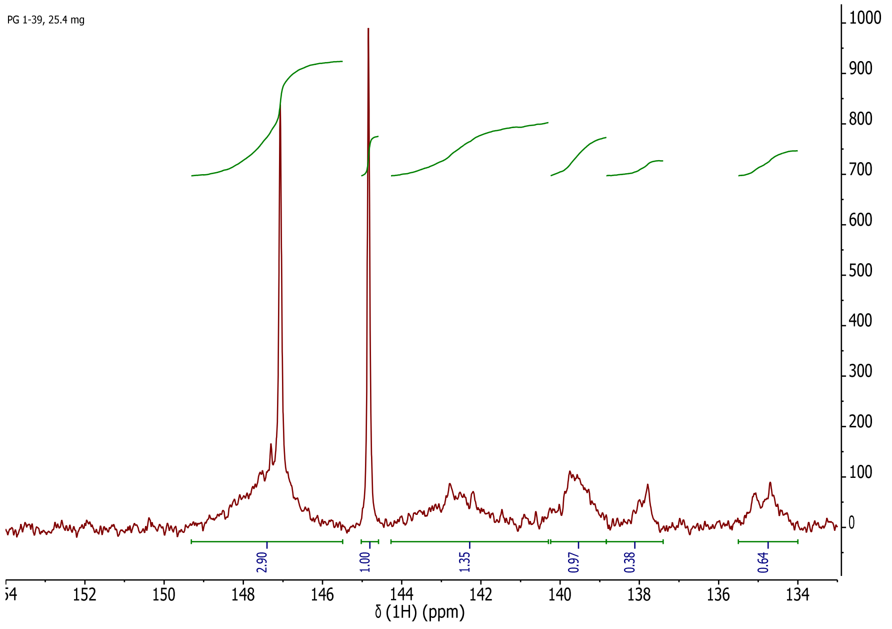

Table S1 (continued): Representative ^{31}P NMR spectra for realised functionalised **WS-OSL** and **WS-OSL**-based copolymer functionalised either chemically or *via* enzymatic catalysis as described in the manuscript.

WS-OSL + $PDMS_{800}$ (10 eq.) [Table 3, entry 4]

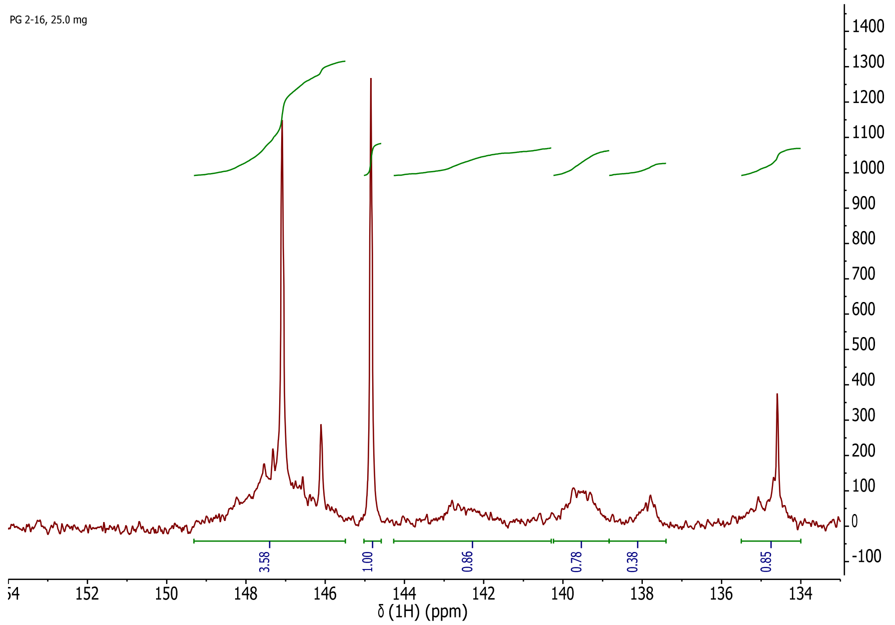

WS-OSL + PEG_{500} (0.1 eq.) [Table 3, entry 5]

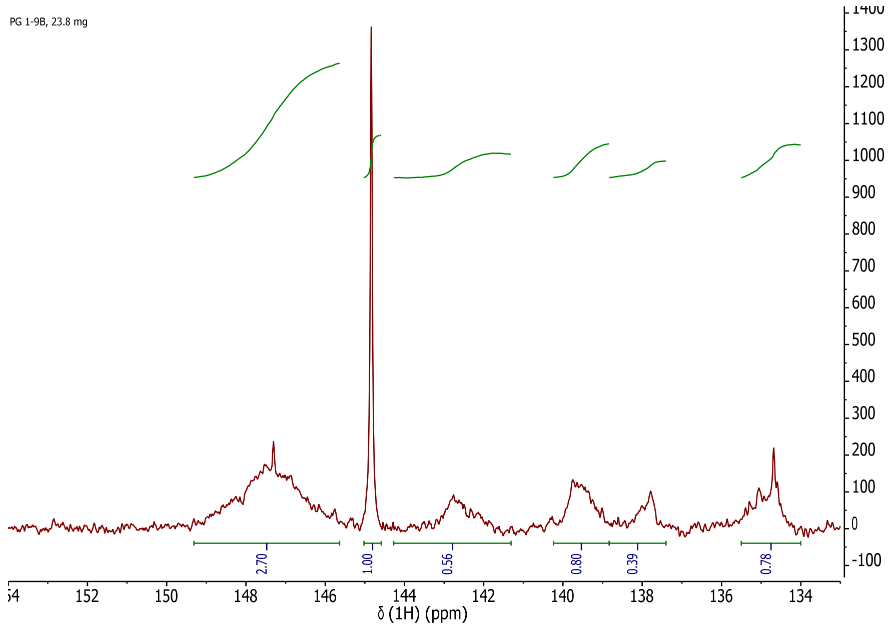

Table S1 (continued): Representative ^{31}P NMR spectra for realised functionalised **WS-OSL** and **WS-OSL**-based copolymer functionalised either chemically or *via* enzymatic catalysis as described in the manuscript.

WS-OSL + PEG$_{500}$ (10 eq.) [Table 3, entry 6]

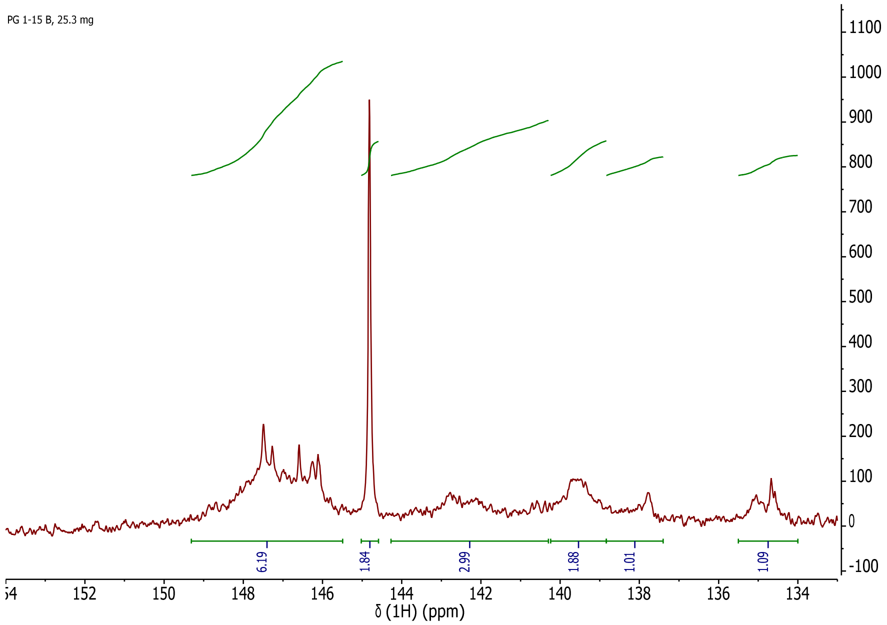

Table S2: Representative ^{31}P NMR spectra for realised functionalised **CS-OSL** and **CS-OSL**-based copolymer functionalised either chemically or *via* enzymatic catalysis as described in the manuscript.

CS-OSL (starting material)

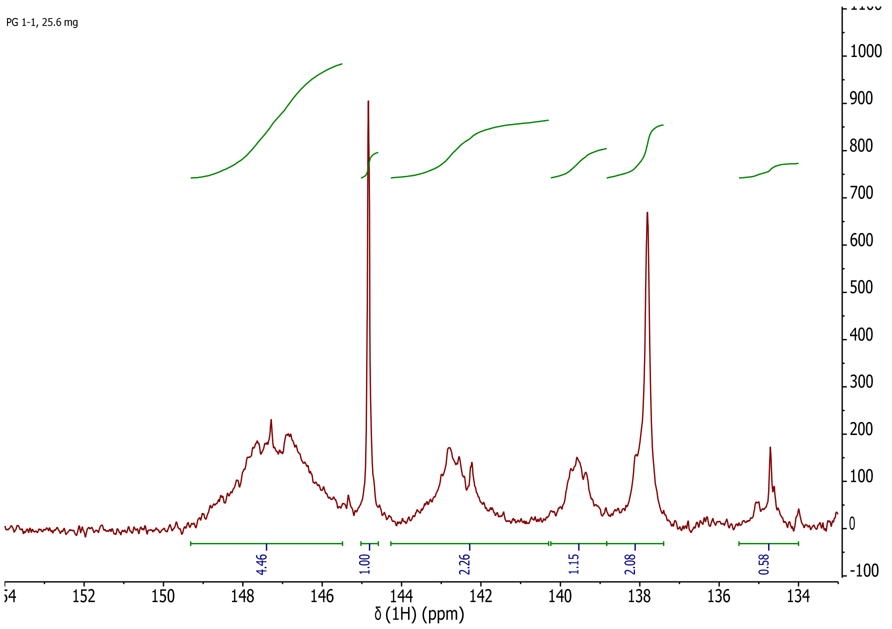

CS-OSL blank

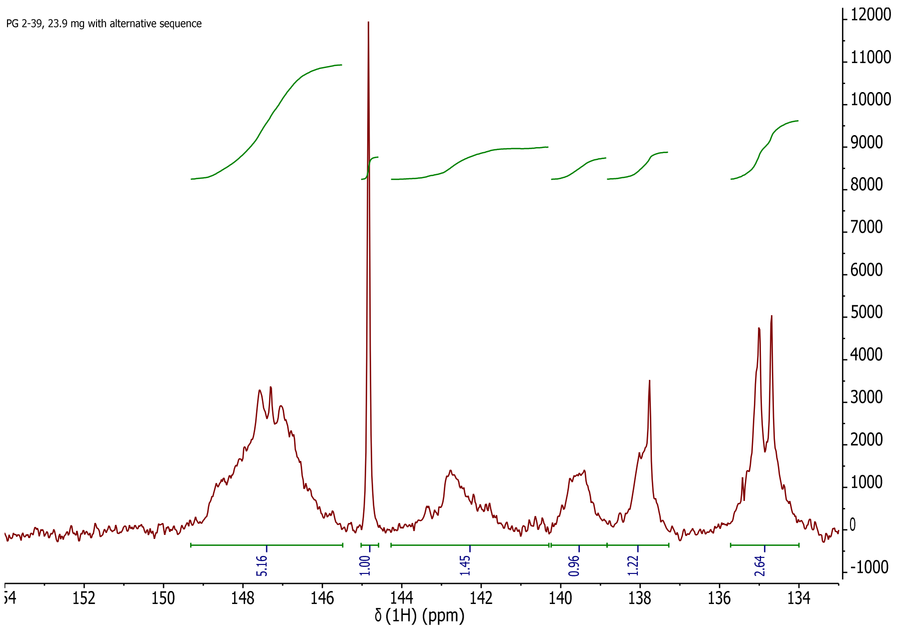

Table S2: Representative ^{31}P NMR spectra for realised functionalised **CS-OSL** and **CS-OSL**-based copolymer functionalised either chemically or *via* enzymatic catalysis as described in the manuscript.

CS-OSL + C_3-NMe$_3$Cl (1.2 eq.) [Table 1, entry 14]

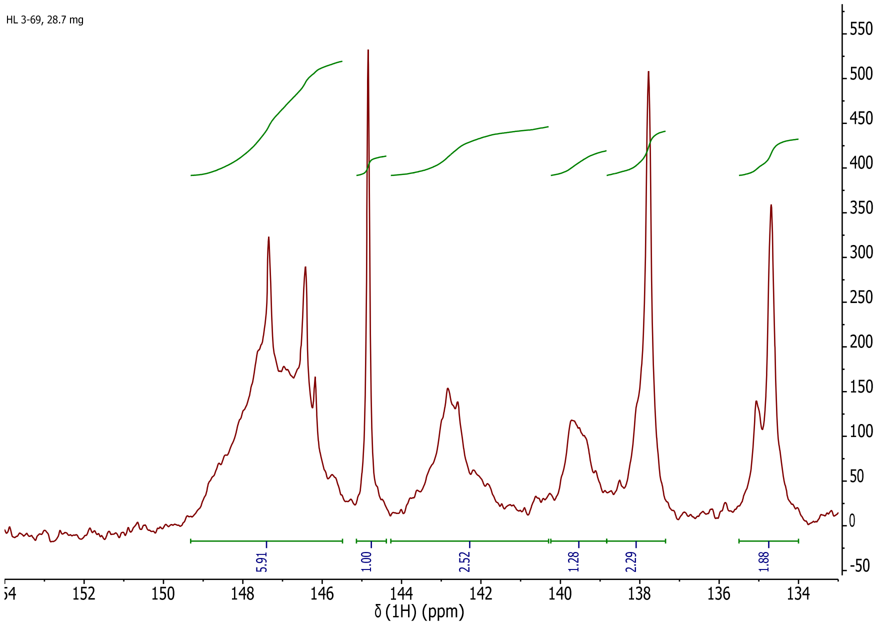

CS-OSL + C_3-CO_2H (1.2 eq.) [Table 1, entry 15]

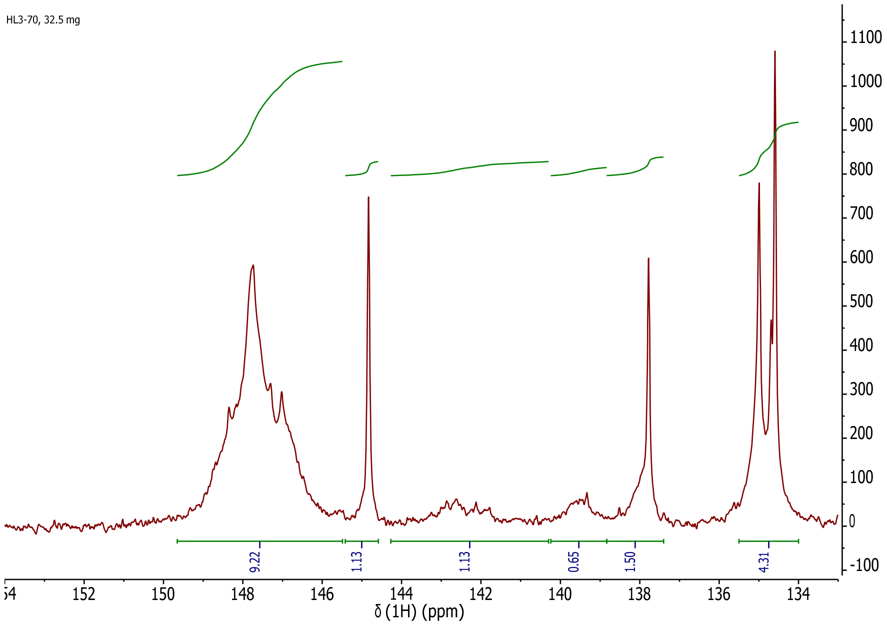

Table S2: Representative ^{31}P NMR spectra for realised functionalised **CS-OSL** and **CS-OSL**-based copolymer functionalised either chemically or *via* enzymatic catalysis as described in the manuscript.

CS-OSL + $PDMS_{5000}$ (1.0 eq.) [Table 2, entry 12]

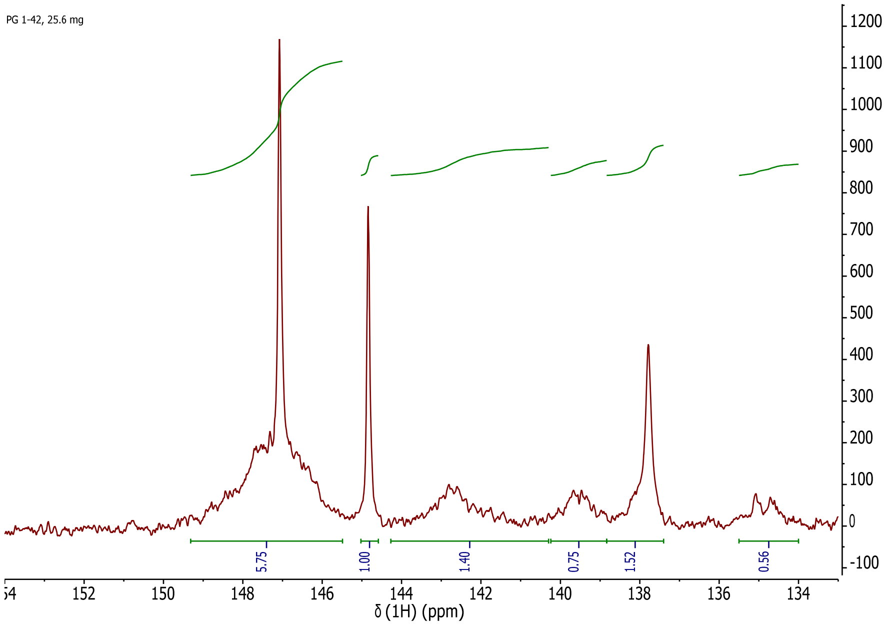

CS-OSL + $PDMS_{800}$ (1.1 eq.) [Table 2, entry 13]

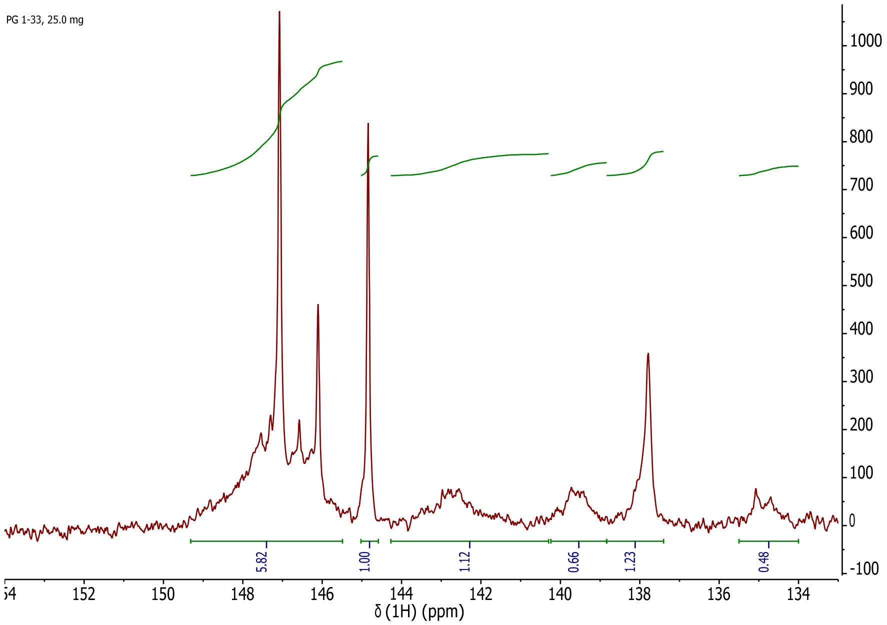

Table S2: Representative ^{31}P NMR spectra for realised functionalised **CS-OSL** and **CS-OSL**-based copolymer functionalised either chemically or *via* enzymatic catalysis as described in the manuscript.

CS-OSL + $PDMS_{5000}$ (1.0 eq.) [Table 3, entry 9]

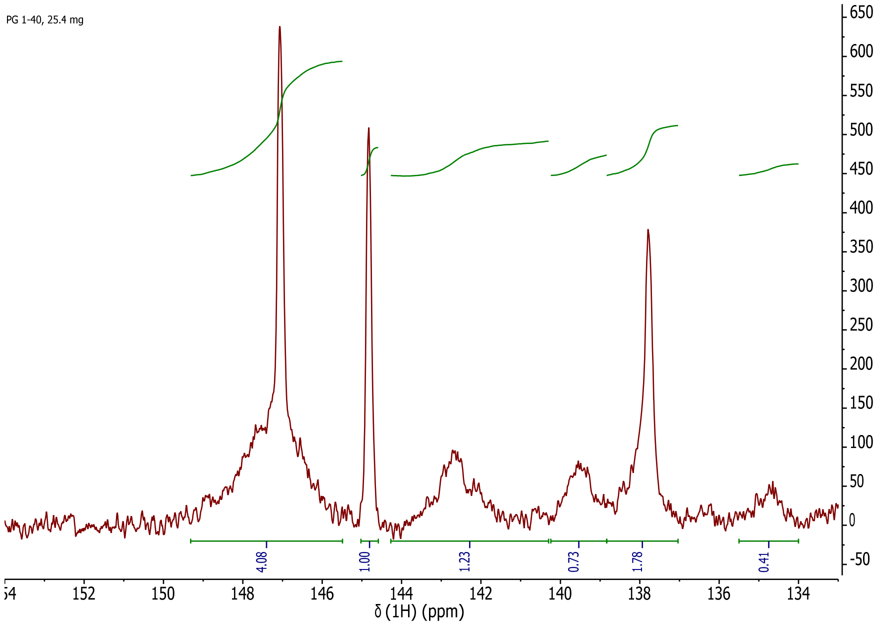

CS-OSL + $PDMS_{800}$ (1.0 eq.) [Table 3, entry 10]

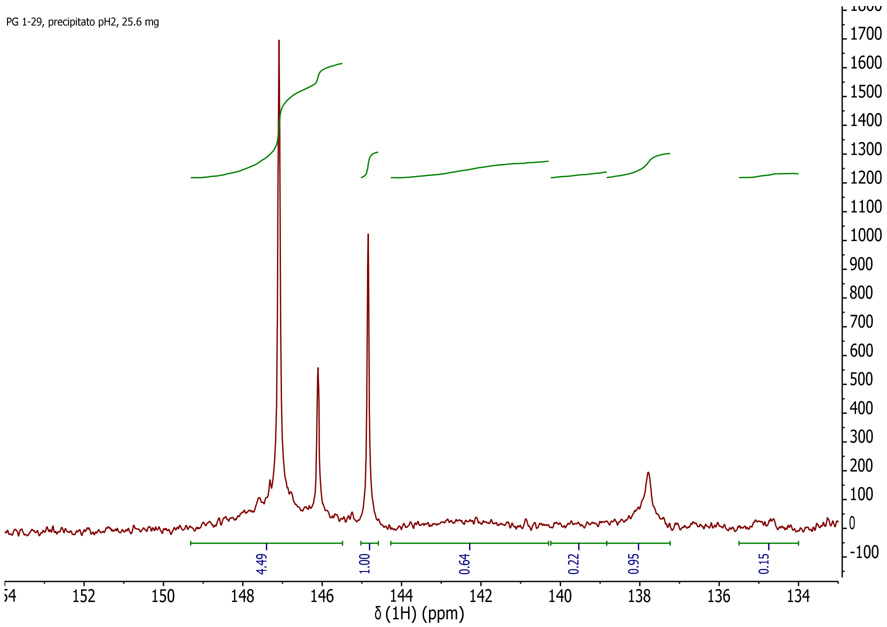

Table S2: Representative ^{31}P NMR spectra for realised functionalised **CS-OSL** and **CS-OSL**-based copolymer functionalised either chemically or *via* enzymatic catalysis as described in the manuscript.

CS-OSL + PEG$_{500}$ (0.1 eq.) [Table 3, entry 11]

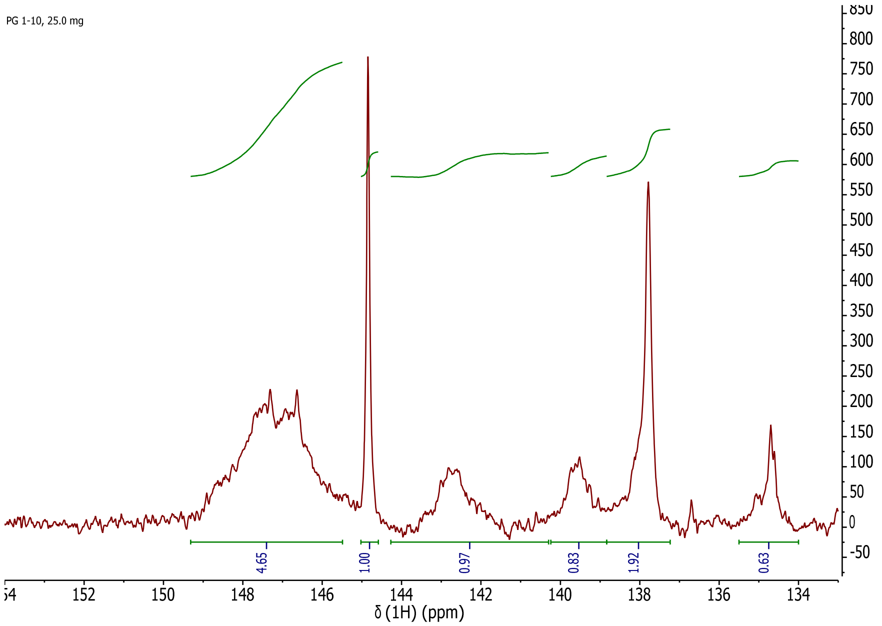

CS-OSL + PEG$_{500}$ (10 eq.) [Table 3, entry 12]

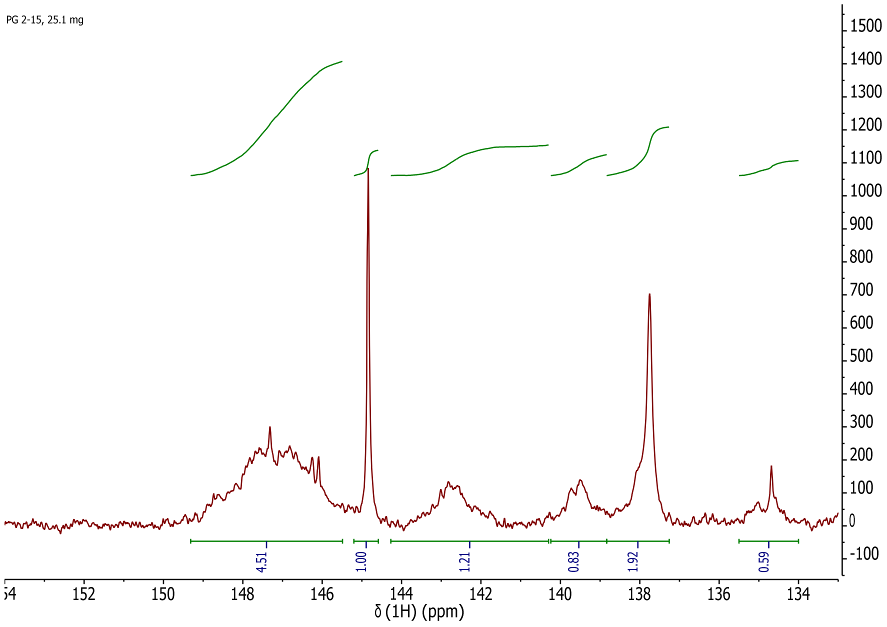

Supplement: Supplementary file 1 — sc0c00886_si_001.pdf [file sc0c00886_si_001.pdf]
